# The structural network of Interleukin-10 and its implications in inflammation and cancer

**DOI:** 10.1186/1471-2164-15-S4-S2

**Published:** 2014-05-20

**Authors:** Ece Saliha Acuner-Ozbabacan, Billur Hatice Engin, Emine Guven-Maiorov, Guray Kuzu, Serena Muratcioglu, Alper Baspinar, Zhong Chen, Carter Van Waes, Attila Gursoy, Ozlem Keskin, Ruth Nussinov

**Affiliations:** 1 Center for Computational Biology and Bioinformatics and College of Engineering, Koc University, Rumelifeneri Yolu, 34450 Sariyer Istanbul, Turkey; 2 Cancer and Inflammation Program, Leidos Biomedical Research, Inc. Frederick National Laboratory for Cancer Research, National Cancer Institute, Frederick, MD 21702, USA; 3 Clinical Genomic Unit, Head and Neck Surgery Branch, National Institute on Deafness and Communication Disorders, NIH, Bethesda, MD 20892, USA; 4 Sackler Inst. of Molecular Medicine, Department of Human Genetics and Molecular Medicine, Sackler School of Medicine, Tel Aviv University, Tel Aviv 69978, Israel

## Abstract

**Background:**

Inflammation has significant roles in all phases of tumor development, including initiation, progression and metastasis. Interleukin-10 (IL-10) is a well-known immuno-modulatory cytokine with an anti-inflammatory activity. Lack of IL-10 allows induction of pro-inflammatory cytokines and hinders anti-tumor immunity, thereby favoring tumor growth. The IL-10 network is among the most important paths linking cancer and inflammation. The simple node-and-edge network representation is useful, but limited, hampering the understanding of the mechanistic details of signaling pathways. Structural networks complete the missing parts, and provide details. The IL-10 structural network may shed light on the mechanisms through which disease-related mutations work and the pathogenesis of malignancies.

**Results:**

Using PRISM (a PRotein Interactions by Structural Matching tool), we constructed the structural network of IL-10, which includes its first and second degree protein neighbor interactions. We predicted the structures of complexes involved in these interactions, thereby enriching the available structural data. In order to reveal the significance of the interactions, we exploited mutations identified in cancer patients, mapping them onto key proteins of this network. We analyzed the effect of these mutations on the interactions, and demonstrated a relation between these and inflammation and cancer. Our results suggest that mutations that disrupt the interactions of IL-10 with its receptors (IL-10RA and IL-10RB) and α2-macroglobulin (A2M) may enhance inflammation and modulate anti-tumor immunity. Likewise, mutations that weaken the A2M-APP (amyloid precursor protein) association may increase the proliferative effect of APP through preventing β-amyloid degradation by the A2M receptor, and mutations that abolish the A2M-Kallikrein-13 (KLK13) interaction may lead to cell proliferation and metastasis through the destructive effect of KLK13 on the extracellular matrix.

**Conclusions:**

Prediction of protein-protein interactions through structural matching can enrich the available cellular pathways. In addition, the structural data of protein complexes suggest how oncogenic mutations influence the interactions and explain their potential impact on IL-10 signaling in cancer and inflammation.

## Background

Inflammation by innate immunity is the first line of defense against pathogenic infections [1]. It is also involved in all phases of cancer development, including tumor initiation, promotion and metastatic dissemination [2-4]. By triggering immunosuppressive mechanisms, inflammation creates a tissue microenvironment which permits tumor growth and metastasis [2]. Inflammatory cells provide growth factors that sustain proliferation, and survival factors that allow escape from apoptosis; it also contributes to extracellular matrix (ECM) modifying enzymes, and to pro-angiogenic factors that facilitate angiogenesis, invasion and ultimately metastasis [3].

Several lines of evidence link cancer and inflammation, emphasizing that chronic inflammation contributes to tumor initiation and progression [5,6]. Chronic inflammatory bowel disease predisposes individuals to colon cancer [6] and individuals with chronic hepatitis are more prone to develop hepatocellular carcinoma [7]. Chronic *Helicobacter pylori *infection and the resulting inflammation is associated with gastric cancer [8]; chronic bronchitis with lung cancer; and pancreatitis with pancreas cancer [9]. Additionally, long term use of non-steroidal anti-inflammatory drugs (NSAIDs) [10] which inhibit pro-inflammatory cytokines, like TNF-α and IL-1β, decrease cancer incidence [11].

Identified in 1989 [12], IL-10 is an anti-inflammatory cytokine that modulates the immune response: if IL-10 is not present or functional, inflammation becomes possible. It restricts the immune response to pathogens and prevents damage to the host. Secreted by immune cells, IL-10 diversely affects cell types in the immune system. Although it terminates inflammatory responses by suppressing monocyte/macrophage function, it also acts as an immunostimulator to promote Th2 response. IL-10 regulates growth and/or differentiation of B cells, NK cells, cytotoxic and helper T cells, mast cells, granulocytes, dendritic cells, keratinocytes, and endothelial cells. Additionally, it stimulates immunoglobulin secretion, and promotes antibody class switching [13]. Therefore, IL-10 has both immune suppressive (anti-inflammatory) and immune stimulatory roles (B and T-cell development).

IL-10 deficiency increases the production of IL-1 (a pro-inflammatory cytokine) and in the absence of IL-10, IL-1 promotes tumor growth in mice [14]. IL-10 also prevents development of regulatory T cells (T_reg_s) and Myeloid-derived suppressor cells (MDSCs) [14-17]. IL-10 deficiency leads to an increase in the number of T_reg_s and MDSCs in tumor tissue. T_reg_s and MDSCs have suppressive roles against tumor-specific immunity that favor tumor growth [4,14,15]. Apart from its anti-inflammatory roles, it is associated with activation of anti-tumor immunity [18]. The presence of IL-10 leads to tumor regression and increase in tumor-specific immunogenicity [19]. In contrast, some studies proposed that blockage of IL-10 signaling promote anti-tumor immunity [20]. These controversial findings stem from the pleiotropic effects of IL-10 and different experimental models (human or animal, *in vitro *or *in vivo*, solid or hematological tumors, presence of exogenous or endogenous IL-10 or IL-10 inhibitors, etc.) and the varying site of IL-10 production [19].

IL-10 is a dimeric cytokine [21] that signals through a tetrameric transmembrane receptor complex, consisting of two IL-10RA (also known as IL-10R1) and two IL-10RB (also known as IL-10R2) proteins [22,23]. Both receptors belong to the class II receptor family, and consist of three domains: an intracellular domain, a transmembrane domain, and an extracellular domain [13]. The receptor complex assembles sequentially: IL-10RA, with higher affinity, binds to IL-10 first and then IL-10RB [21]. IL-10 binding to the extracellular domain of IL-10RA leads to phosphorylation of JAK1 (Janus Kinase-1) and TYK2 (Tyrosine Kinase-2), that interacts with IL-10RA and IL-10RB [24], respectively. Specific tyrosine residues on the intracellular domain of the IL-10RA are then phosphorylated by these kinases. The STAT3 (Signal Transducer and Activator of Transcription-3) transcription factor binds to those tyrosine residues and gets phosphorylated. Activated STAT3 then translocates to the nucleus as a homodimer, and activates transcription of anti-apoptotic and cell-cycle-progression genes [25,26].

IL-10 has many functional partners, one of which is the α_2_-macroglobulin (A2M). IL-10 forms a stable complex with activated A2M [27]. A2M is a large homotetrameric glycoprotein [28] in the plasma, and in the extracellular space. It is a proteinase inhibitor, and has a peptide stretch, called the 'bait region'. Cleavage of the bait region by a proteinase leads to a conformational change in the protein that causes proteinase trapping, and receptor-mediated endocytosis of the A2M-proteinase complex [29]. A2M is also a cytokine transporter. Many cytokines, including IL-10, and growth factors bind to A2M non-covalently *in vivo *[30]. When A2M forms complexes with IL-10, TGF-β (anti-inflammatory cytokines) and IFN-γ, it accelerates the appearance of these cytokines in the blood [31,32]. A2M in its native form increases the half-life of bound cytokines in the plasma by protecting them from proteolysis [30]. Thus, at sites of inflammation, A2M concentration rises as a response to an increase in proteinase level. [27]. A2M also contributes to the anti-inflammatory response of IL-10 by preventing its destruction.

IL-10 and IL-10Rs are able to interact with many partner molecules in the signaling network; however, their detailed protein structural interactions, as well the corresponding mutational mechanisms have not been well illustrated. In this study, we constructed the structural pathway based on protein-protein interactions (PPIs). The commonly used node-and-edge description of pathways, where nodes represent proteins and edges the interactions between them, are useful, but do not provide structural interaction detail [33,34]. Further, in many cases, such as IL-10 and the receptors in this study, the available structural interaction data of the proteins are incomplete. However, recently developed computational structural approaches, such as PRISM (PRotein Interactions by Structural Matching tool), are capable of predicting PPI and can help filling in the gaps. PRISM [35,36] is a motif-based protein-protein interaction prediction tool which uses a knowledge-based strategy to construct and analyze structural PPI networks. PRISM is based on the notion that evolution has exploited favorable structural motifs adapting them to different functions, in protein folds and at protein-protein-interfaces [37-39], lending robustness to its predictions. PRISM has predicted protein interactions successfully [40,41] for different pathways, like apoptosis [42], ubiquitination [43], MAPK [41,44], the Toll-like receptor pathways, [1] and for identifying drug off-targets [45]. The success of PRISM is very close to %100 (87 out of 88 cases) in rigid-body prediction [40]. Recently we have further enhanced it by introducing ensemble docking, by exploiting different conformations, and PRISM could predict two thirds of the 'difficult' cases of a docking benchmark dataset [41]. Here, we applied this enhanced PRISM protocol to construct the structural protein-protein interaction (PPI) network of IL-10 centered signaling. Importantly, the analysis was able to identify mutations falling in the interfaces and to predict their effects on interactions such as of IL-10 with its receptors, IL-10 with A2M, and A2M with APP and KLK13. This allowed us to enrich the structural interaction data of IL-10 with its partners, and to analyze the mechanisms of mutations that lead to inflammation, and cancer through their impact on predicted interactions.

## Methods

### Reconstruction of the structural PPI network of IL-10 centered signaling

We used the String server [46] for selecting the first and second-degree neighbors of IL-10. Only interactions with experimental evidence and confidence score larger than 0.4 (the default confidence value) were considered. There were 4 first-degree and 45 second-degree neighbor proteins of IL-10 (Additional file 1). Overall, there were 50 proteins comprising the IL-10 centered protein-protein interaction network.

We used PRISM [35,36] for modeling protein-protein interactions in the IL-10 centered network. PRISM searches for the motifs on the target protein surfaces similar to known interactions considering both geometrical complementarities and evolutionary conservation of hot spots. It treats proteins with at least 15 residues. To model an interface, PRISM requires the 3D structures of the proteins of interest (for further details of the PRISM protocol, see [36]). 39 of these 50 proteins have structural data in the PDB (corresponding to 958 PDB chains) and we could build homology models for the remaining 10. IGHV3-6's sequence information could not be found, so this protein is not included in our analysis (Additional file 1 Table S1). The I-TASSER server [47] was used for homology modeling and the top 5 models generated by the server were included in the predictions.

We reduced the redundancy of similar interface architectures for each protein, using TM (template modeling)-align [48]. PDB structures having TM-scores larger than 0.5 and RMSD under 2.5Å were classified. Then, a representative PDB structure was assigned for each similar structure group and we ended up with 127 representative structures for 39 target proteins. The final IL-10 centered network is composed of 49 proteins and 70 interactions (Additional file 1 and 2).

### Mapping oncogenic mutations onto the interfaces of predicted protein-protein complexes and *in silico *mutagenesis

**Figure 1:**
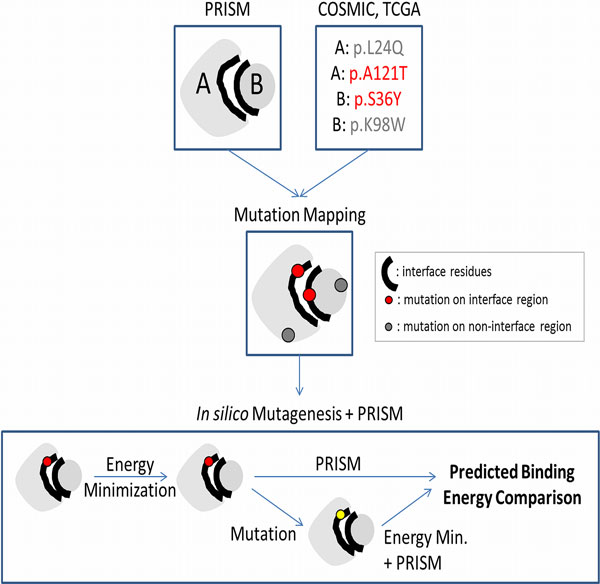
**Mapping oncogenic mutations onto the interfaces of predicted protein-protein complexes and *in silico *mutagenesis**. Mutational data related to the proteins in the network were taken from the COSMIC database [49,50] and The Cancer Genome Atlas, TCGA [51]. To map oncogenic mutations, we identified the interfaces of the modeled protein-protein complexes using the HotPoint web server [52]. After the mutational mapping, we performed *in silico *mutagenesis to observe the effects of mutations - that are on the interface - on the interactions. We computationally mutated those key residues using the FoldX plugin [53] for the YASARA molecular viewer [54]. We minimized the energies of the proteins both before and after the mutation and then used the mutant structures to re-run PRISM [35,36] and model the new interaction between the mutant target and its partner.

The reconstructed structural PPI network of IL-10 centered signaling not only reveals many important details about the mechanism of protein-protein interactions but also offers the possibility of observing the effects of oncogenic mutations. In the case studies presented below, mutational data related to the proteins in the network were taken from the COSMIC (Catalogue of Somatic Mutations in Cancer) database [49,50] and cBioPortal for Cancer Genomics (The Cancer Genome Atlas, TCGA) [51]. To map oncogenic mutations, we identified the interfaces of the modeled protein-protein complexes using the HotPoint web server, which uses conservation, solvent accessibility and pairwise residue potential data to determine computational hot spots [52]. After the mutational mapping, we performed *in silico *mutagenesis to observe the effects of the mutations on the interactions (Figure 1). We computationally mutated those key residues using the FoldX plugin [53] for the YASARA molecular viewer [54]. We minimized the energies of the proteins both before and after the mutation and then used the mutant structures to re-run PRISM [35,36] and model the new interaction between the mutant target and its partner (Figure 1).

## Results and discussion

**Figure 2:**
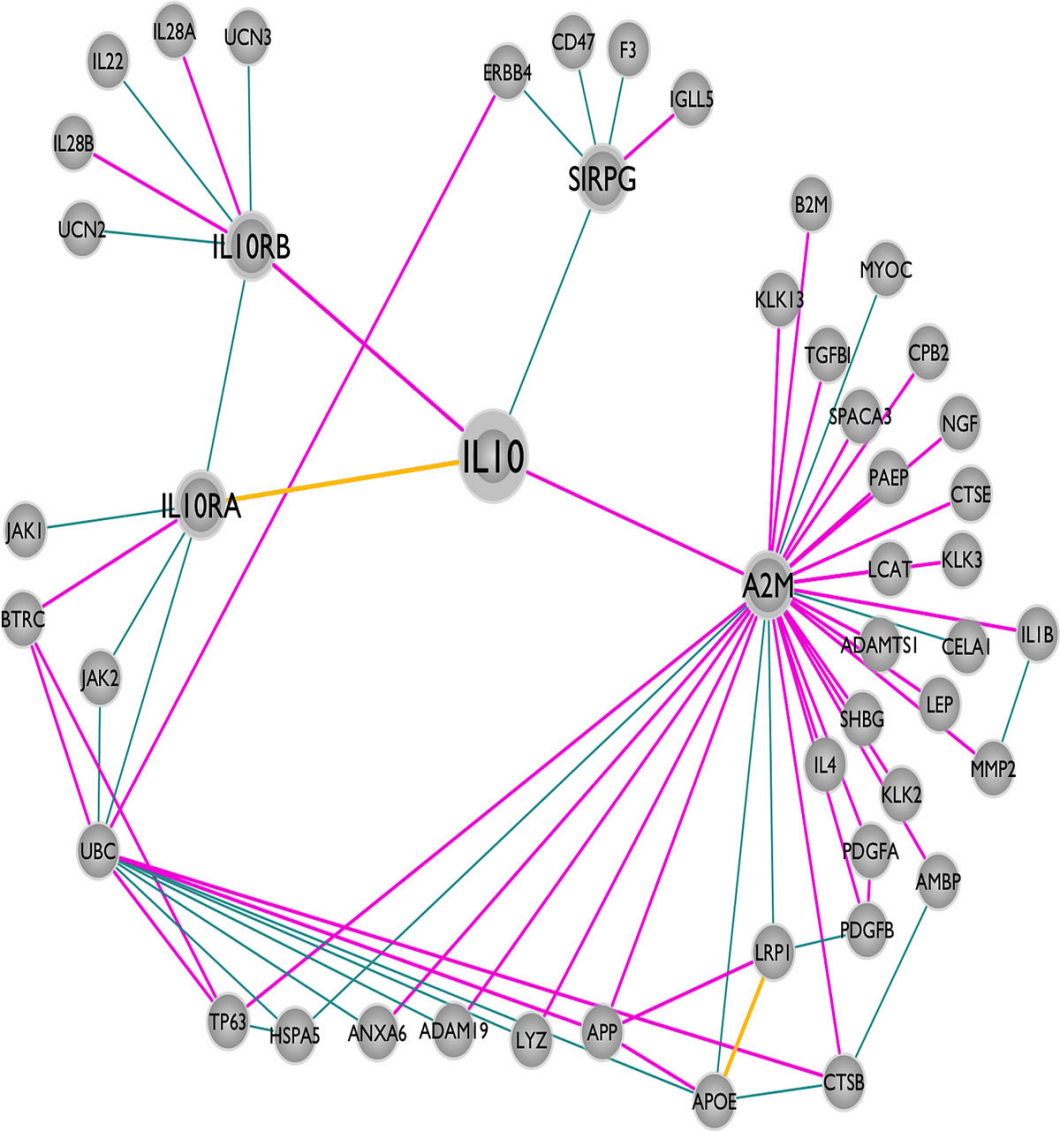
**The protein-protein interaction network of Interleukin-10**. There are 49 proteins and 70 interactions in this network and only 2 of the interactions have structural data in a complex form in the PDB (edges highlighted in yellow). We modeled the interfaces for 40 additional interactions. Thus there are 42 interactions with interface models (edges highlighted in pink). The remaining 28 edges (out of 70) could not be modeled and are shown in cyan.

We constructed the IL-10 centered human structural protein-protein interaction network with first and second-degree protein neighbors. This network is composed of 49 proteins and 70 interactions between them (Figure 2). Among these 70 interactions, the structure of only two (IL-10 - IL-10RA and APOE - LRP1) are deposited in the PDB in a complex form. By using PRISM, we predicted the structures of the PPI interfaces and 40 additional interactions were structurally modeled (Figure 2). As a result, the available structural data increased from 2 to 42.

**Figure 3:**
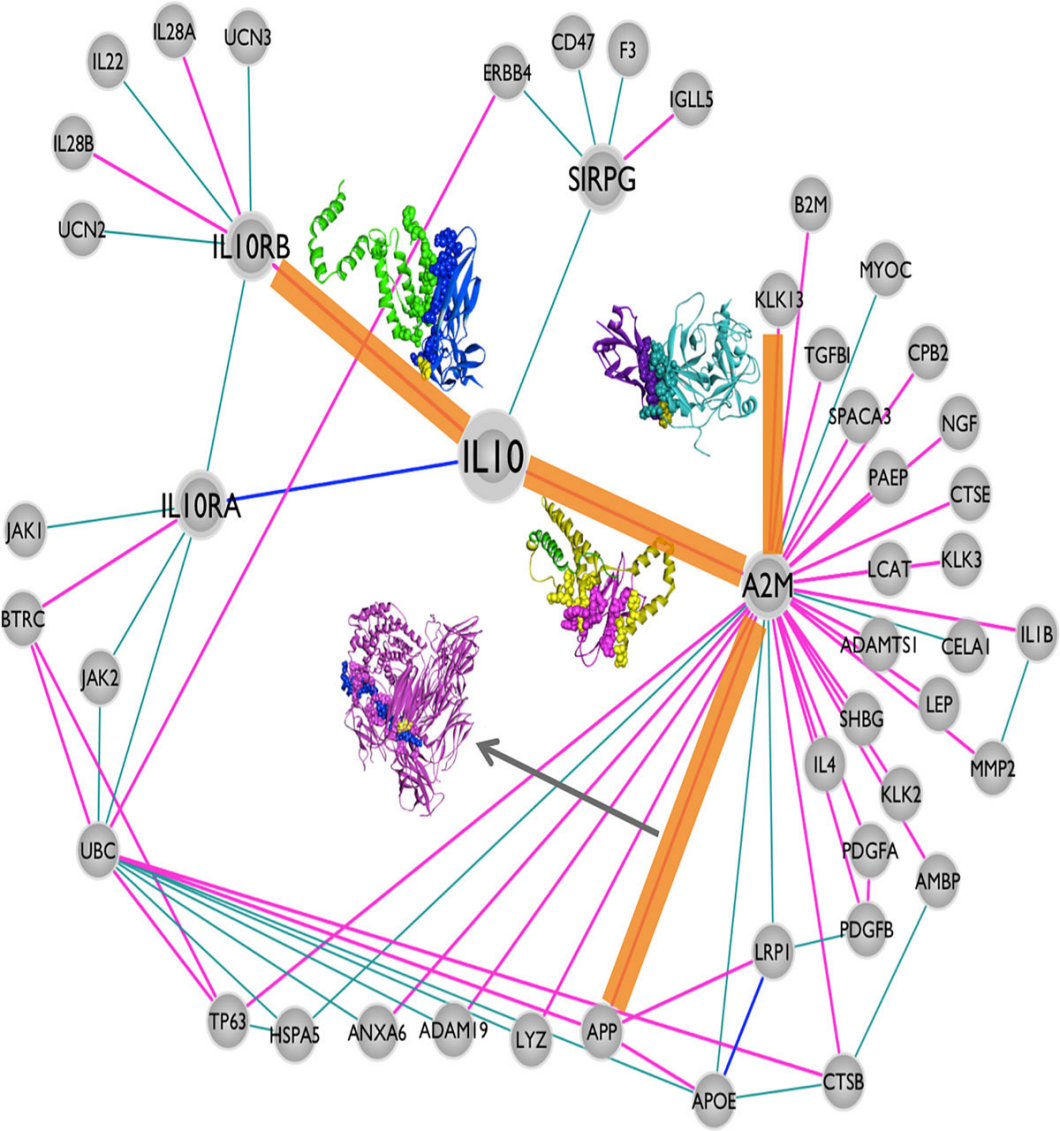
**The case study interactions highlighted on IL-10 centered network**. The thick orange lines indicate four case studies explaining the relation between mutations on the interfaces of protein-protein complex models and cancer (IL10-IL10RB, IL10-A2M, A2M-KLK13 and A2M-APP).

In mutation databases, like COSMIC [50,55], there are thousands of experimentally verified cancer mutations but the precise mechanisms of mutations, how they change protein functions and contribute to cancer pathogenesis is unknown [56]. Proteins function through interactions and mutations that disrupt interactions also change protein functions. Some mutations abolish protein interactions, whereas others make the interactions stronger or change protein folding, favoring aggregation of proteins, as in the case of formation of amyloids in Alzheimer's disease [57]. 4% of all mutations in the databases were computationally predicted to be related with protein interactions [56]. Structural knowledge of protein-protein interfaces is important, allowing mapping mutations and SNPs onto the structures of the complexes, identifying those falling on the interfaces, and predicting their effects on protein interactions [33,58-60]. PRISM is a tool that provides the opportunity to observe the effects of these mutations on the interactions. Overall, 879 "missense" and "coding silent" mutations from COSMIC database [49,50] are mapped onto 29 target proteins (40 target PDB structures) in the IL-10 centered network (Table 1 and Additional file 3). Each of the interactions of these 29 target proteins can be studied for observing the effects of mutations that are on the interfaces of the predicted models. Below, we present four examples of interactions in the reconstructed structural IL-10 centered network and these interactions are related to oncogenesis (Figure 3). In these case studies, we computationally mutated the wild type residues to their corresponding oncogenic mutation variants and analyzed the effects on the original interaction.

**Table 1:** The distribution of COSMIC "missense" and "coding silent" mutations mapped onto the target structures in IL-10 centered network.

Protein	PDB	Mutation Number	Protein	PDB	Mutation Number
A2M	2p9rA	16	IL10	2ilkA	12

A2M	1bv8A	7	IL10RA	1lqsR	25

A2M	4acqC	139	IL10RB	3lqmA	24

ADAMTS1	2jihB	34	IL1B	3ltqA	16

AMBP	4es7A	12	IL28B	3hhcB	21

ANXA6	1m9iA	35	IL4	1bbnA	9

APOE	2kc3A	5	KLK3	2zchP	29

APOE	2l7bA	8	LEP	1ax8A	11

APP	1tknA	13	LRP1	2knyA	7

APP	3ktmE	9	LYZ	1lz6A	6

APP	2llmA	5	MMP2	3ayuA	20

APP	3nylA	21	NGF	1wwwW	10

APP	1owtA	6	PDGFA	3mjkA	6

APP	3umkA	20	SHBG	1kdkA	6

B2M	1ypzB	22	SIRPG	2jjwA	12

B2M	3ov6A	29	TGFBI	1x3bA	15

BTRC	1p22A	30	TP63	2y9tA	8

CPB2	3d68A	33	TP63	2rmnA	29

CTSB	3pbhA	22	TP63	4a9zC	5

ERBB4	2ahxB	121	UBC	3b0aD	21

## Case studies

### The relation between the interaction of IL-10 with its receptors and the implications in inflammation & cancer

**Figure 4:**
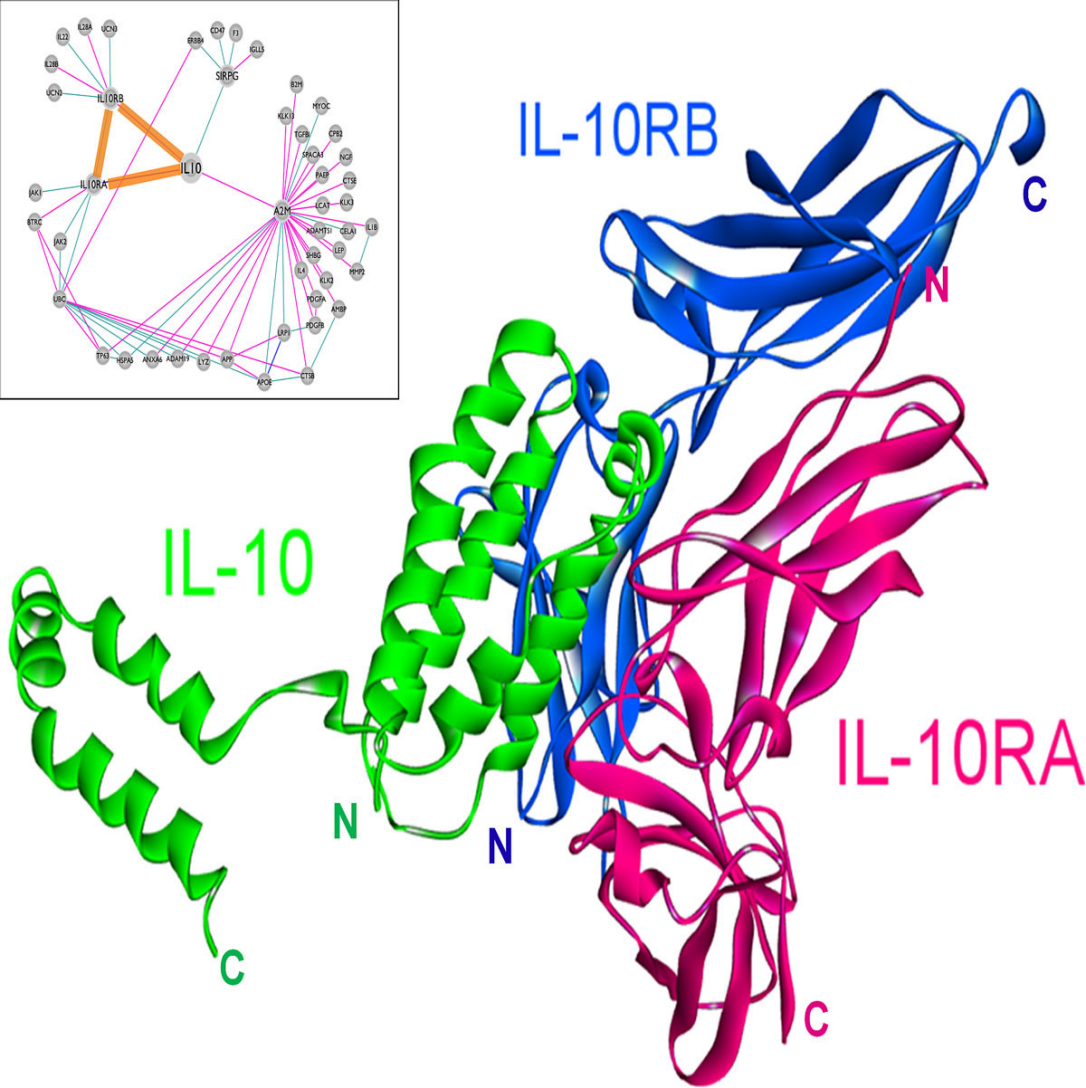
**The predicted complex structure of IL-10 with its receptors, IL-10RA and IL-10RB**. The complex between IL-10 and IL-10RA is available in PDB (PDB ID: 1j7v, L and R chains, respectively) and the interaction of IL-10 (PDB ID: 2ilkA) with IL-10RB (PDB ID: 3lqmA) is modeled by PRISM. Receptors bind to different surfaces of IL-10 that are close to each other.

The structure of the IL-10 and IL-10RA complex is available in the PDB [21], whereas the IL-10 and IL-10RB complex structure has not been solved yet. We were able to model the interaction of IL-10 (PDB code: 2ilkA) with IL-10RB (3lqmA) using PRISM (Figure 4). The template interface used in the prediction of this interaction is 2a6aAB (a glycoprotein endopeptidase homodimer) and the interaction energy score is -15.98 energy units. In order to confirm our model experimentally, we compared the interface of the predicted structure with the critical residues in the binding of IL-10 - IL-10R2 (IL-10RB) [61], which were determined by surface plasmon resonance and cell-based assays. There is a good agreement between the predicted interface residues and experimentally determined critical residues in binding assays. Five out of six residues, Asn21, Arg24, Arg32, His90 and Ser93, which are determined to be critical in binding, are also predicted to be in the interface. In order to execute its function, IL-10 needs to interact with both receptors IL-10RA and IL-10RB at the same time, as the receptor complex is a ternary structure [61]. In the PRISM-predicted model, the receptors can bind to IL-10 without clashing and can therefore form a ternary complex in agreement with functional data (Figure 4). PRISM also predicts the interaction of IL-10 with IL-10RA with -25.54 energy units, utilizing its own complex structure as the template interface (1j7vLR). IL-10RA has higher affinity for IL-10 (-25.54 energy units) compared to the affinity of IL-10RB for IL-10 (-15.98 energy units). This is also in line with the sequential mechanism of the ternary complex formation with IL-10 binding first to IL-10RA, and then IL-10RB is recruited [61].

**Figure 5:**
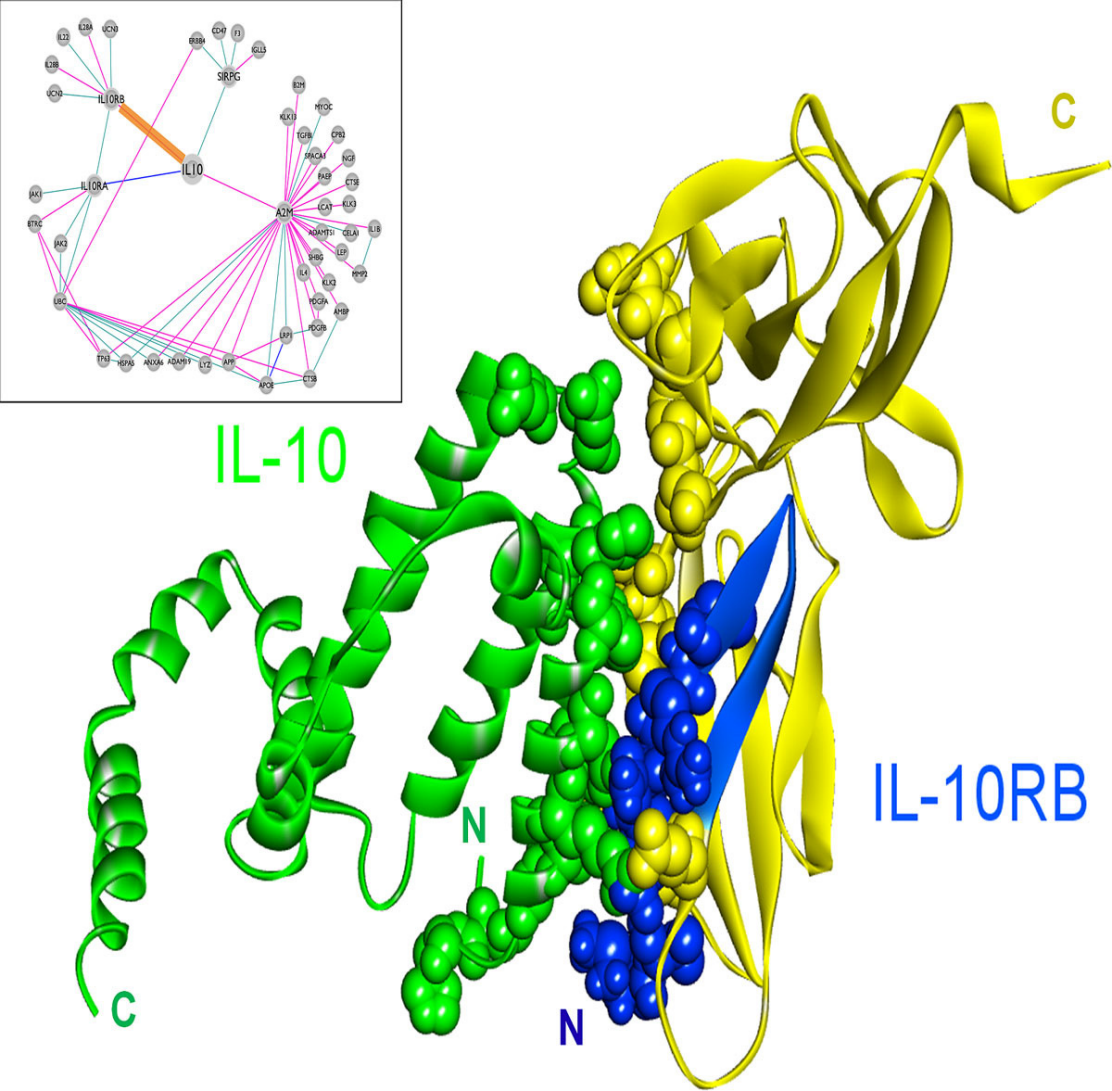
E41* nonsense mutation on IL-10RB protein. IL-10 (2ilkA) is shown in green, blue protein is IL-10RB (3lqmA) and the yellow segment is the deleted part in E41* nonsense mutant of IL-10RB. This mutation causes the loss of a large part of the protein, including most of the interface between IL-10 and IL-10RB, so that it blocks the interaction of IL-10RB with IL-10. If IL-10 cannot interact with its receptors, downstream signaling does not take place and anti-inflammatory outcomes of IL-10 pathway will be lost, allowing inflammation to develop.

**Figure 6:**
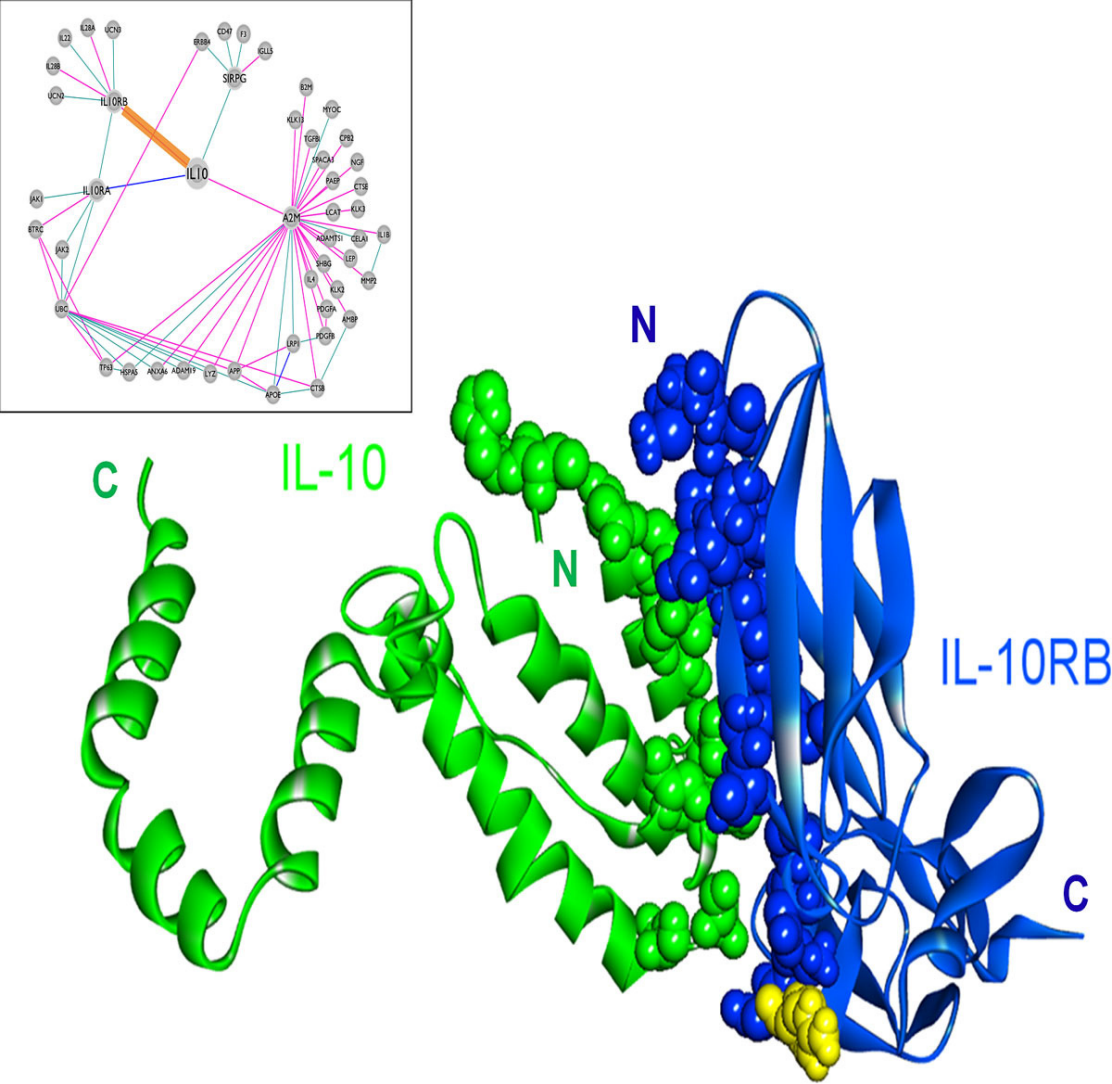
**R198W substitution mutation on IL-10RB protein**. IL-10 (2ilkA) is shown in green, blue protein is IL-10RB (3lqmA). R198, highlighted in yellow, is the substituted residue on the mutant IL-10RB. This mutation is very close to the interface between IL-10 and IL-10RB and it abolishes the interaction of IL-10RB with IL-10.

Structural details in interactions of IL-10 with its receptors may shed light on the mechanisms of mutations. The role of IL-10 in cancer was shown by Tanikawa et al. that IL-10 deficiency causes a rise in the production of IL-1, a pro-inflammatory cytokine, which in turn leads to increased tumor growth in mice [14]. Mutations in IL-10 or its receptors may disrupt their interactions, thereby preventing IL-10 signaling. For instance, E41* nonsense mutation in IL-10RB, abolishes the interaction of IL-10 with IL-10RB, blocking IL-10 signaling. This mutation causes loss of most of the IL-10RB, including a large portion of the interface between IL-10 and IL-10RB (Figure 5, the part drawn in yellow, Table 2). According to the TCGA data, this mutation is observed in lung adenocarcinoma with 2% frequency [51]. Furthermore, the R198W substitution mutation in IL-10RB (from the COSMIC database [49,50]), also destroys the interaction between IL-10 and IL-10RB (Table 2), and this mutation is observed in endometrioid carcinoma. Importantly, Figure 6 shows that the R198W mutation in IL-10RB falls right next to the interface. Blockage of IL-10 signaling may lead to enhanced inflammation and increased number of T_reg_s and MDSCs, which inhibit tumor immunity, allowing tumors to grow [15].

**Table 2:** Comparison of the binding energy scores for wild type and mutant structures

Target Structures		Binding Energy Score
IL-10 (1j7vL) (*wt*)	IL-10RA (1j7vR) (*wt*)	-25.54

IL-10 (2ilkA) (*wt*)	IL-10RB (3lqmA) (*wt*)	-15.98

IL-10 (2ilkA) (*wt*)	IL-10RB (3lqmA) (R198W)	-6.09

IL-10 (2ilkA) (*wt*)	IL-10RB (3lqmA) (E41*)	N/A

IL-10 (2ilkA) (*wt*)	A2M (1bv8) (*wt*)	-39.2

IL-10 (2ilkA) (Q56*)	A2M (1bv8) (*wt*)	N/A

A2M (4acqC) *(wt)*	APP (2llmA) *(wt)*	-39.12

A2M (4acqC) (R945Q)	APP (2llmA) *(wt)*	-9.12

hK13 (model) (*wt*)	A2M ( 1bv8A) (*wt*)	-65.22

hK13 (model) (R236L)	A2M ( 1bv8A) (*wt*)	+19.48

### The association of IL-10 with A2M and its implications for inflammation and cancer

**Figure 7:**
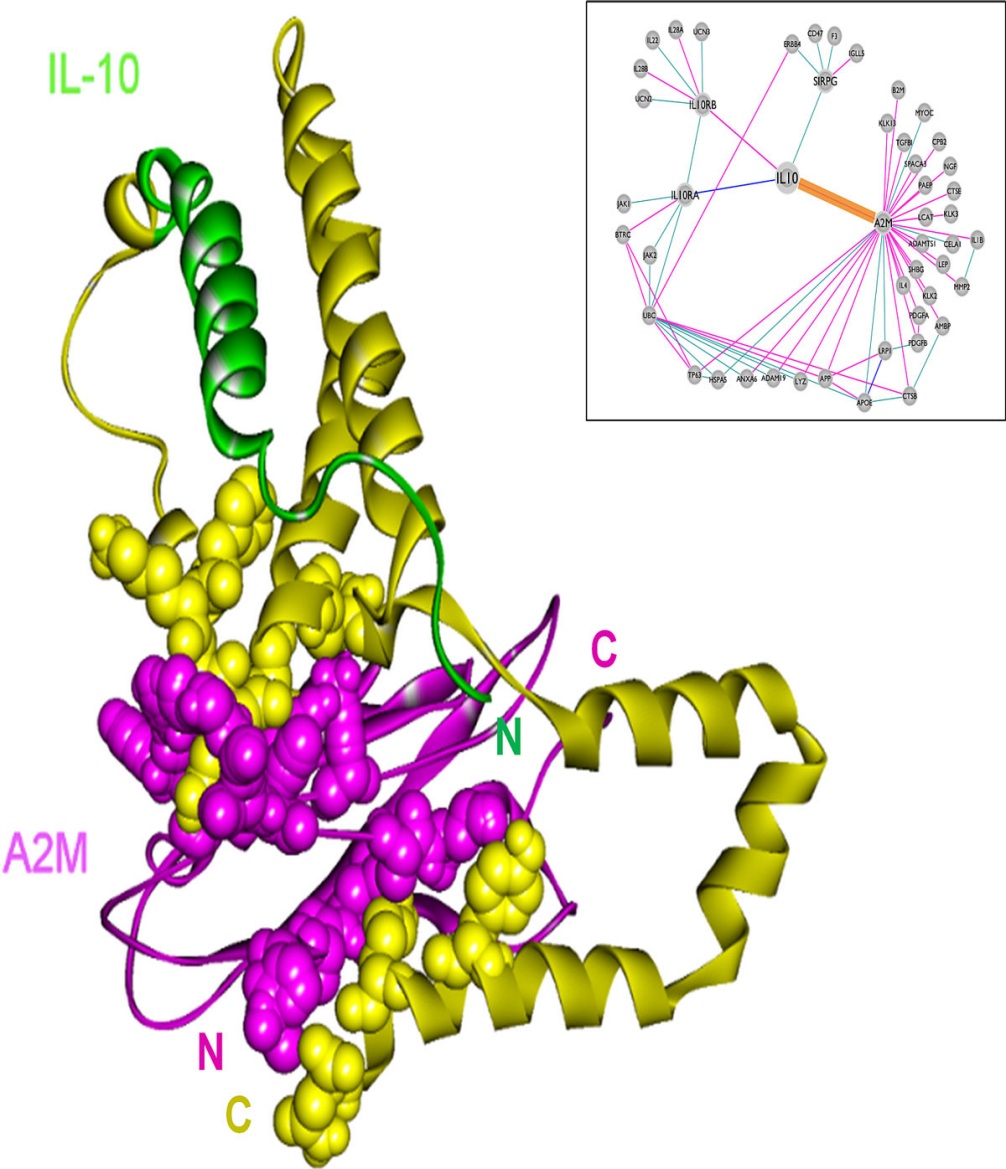
**IL-10 (2ilk, green) is predicted to bind to A2M (1bv8, purple) with a binding energy score of -39.2**. The template interface is 1mwqAB, a hypothetical protein from *H. influenzae *with a putative active-site phosphohistidine. The yellow segment is the deleted part of IL-10 due to Q56* nonsense mutation. The deleted part includes the interface region, so the interaction between IL-10 and A2M is disrupted.

Here we concentrate on the interaction of IL-10 with α_2_-macroglobulin (A2M). A2M mediates the inflammatory response through acting as a cytokine transporter. A2M binding to IL-10 facilitates the recruitment of this cytokine to the site of inflammation and triggers an anti-inflammatory response [31,32]. A2M also protects IL-10 from proteolysis [30]. Thus, if the interaction between these two is disrupted, inflammation will occur [31,32], which may favor cancer development. Normally, IL-10 is predicted to bind to A2M with a binding energy score of -39.2 (Figure 7). We mutated IL-10 computationally based on the oncogenic mutation to see whether the interaction is affected. Q56* nonsense mutation in IL-10, that has been seen in lung adenocarcinoma [50,51], is observed to abolish the association of IL-10 with A2M. As can be seen from Figure 7, this nonsense mutation causes the complete loss of the interface between IL-10 and A2M (the yellow labeled part).

### The interaction between A2M with APP and its implications in cancer

Here, we focus on the interaction between A2M, one of the interaction partners of IL-10, and β-amyloid precursor protein (APP). Extracellular cleavage of APP produces C99, a cell membrane-bound fragment, which is further cleaved by γ-secretase, and releases the intracellular domain of APP to produce amyloid-β (Ab) [62]. Ab forms a complex with native A2M and the complex is internalized by the A2M receptor, a low density lipoprotein receptor-related protein (LRP), and degraded [63].. According to the PRISM results, A2M (PDB ID: 4acqC, residues 24-1474) forms a stable complex with APP (PDB ID: 2llmA, residues 686-726) with a binding energy score of -39.12. The template interface used in the prediction is 1fftAC (ubiquinol oxidase from *Escherichia coli*). APP is an integral type I transmembrane protein with a single transmembrane domain (residues 700-723), a large extracellular ectodomain (residues 18-699), and a short cytoplasmic tail (residues 724-770) [62].

**Figure 8:**
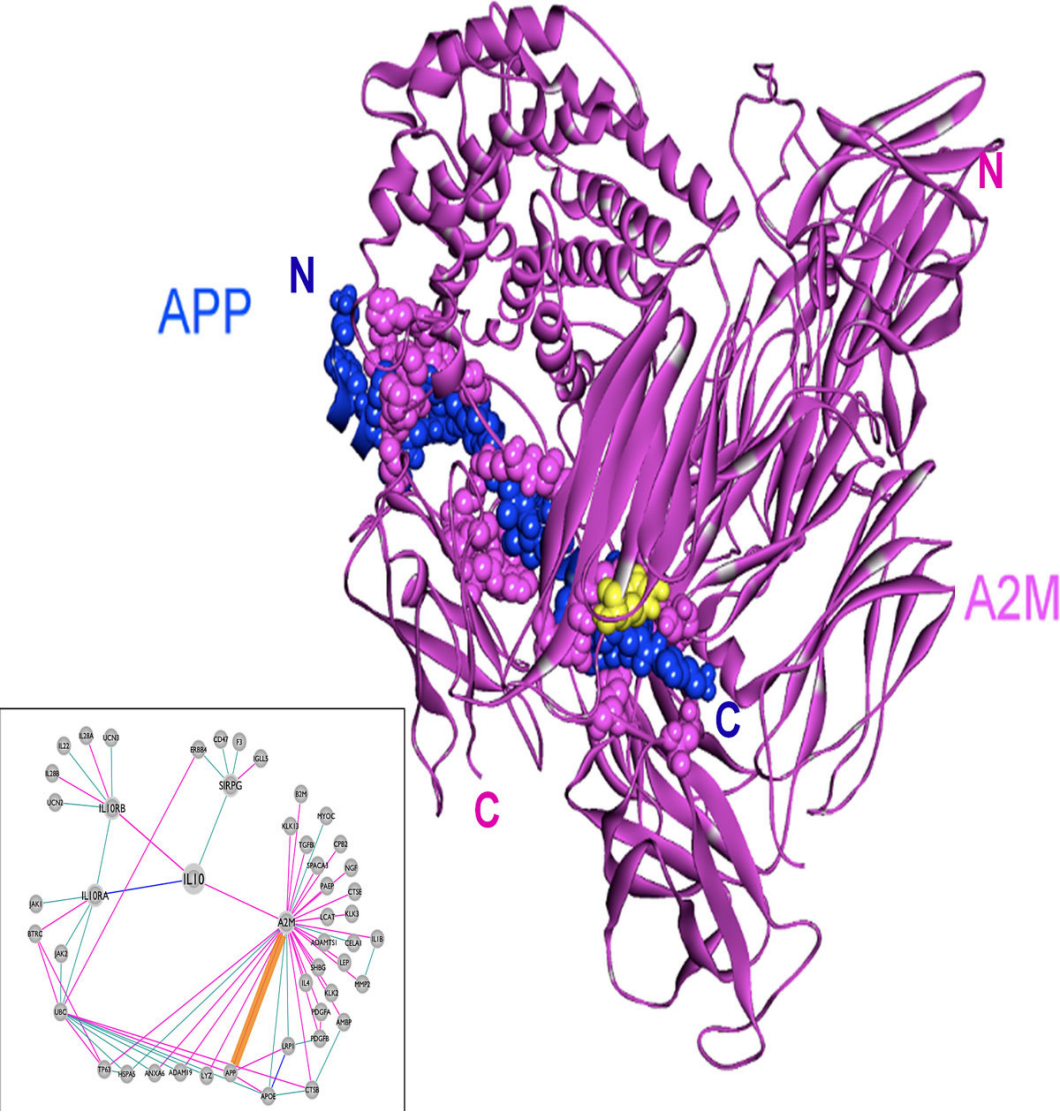
**The predicted complex structure of A2M (4acqC, purple) and APP (2llmA, blue) with an energy score of -39.12**. Template interface: 1fftAC (an ubiquinol oxidase). R945, highlighted in yellow, is the mutated residue. The predicted complex structure of mutated A2M (4acqC, purple) and *wt *APP (2llmA, blue) has an energy score of -9.12.

APP has been found to be up-regulated in many cancers, including pancreatic [65], colon [66], and prostate cancer [67] and squamous cell carcinoma [65]. Gain-of-function studies indicated that overexpression of APP causes increased cell proliferation [67,68]. We performed *in silico *mutational analysis in order to observe the effect of the mutations on the A2M - APP interaction, and found a possible association between abrogation of this interaction and cancer. Mutations mapping to the interface of the predicted complex were identified, and a missense mutation, R945Q, which is observed in colorectal cancer was selected for further analysis [50,51]. R945 in human A2M is a hot spot interface residue (Figure 8) and mutating this residue to glutamine resulted in a noticeable change in the binding energy score (Table 2). The score for the mutated structure decreased to -9.12 indicating that substituting arginine with glutamine greatly weakens the interaction. This observation suggests that the weakened A2M - APP interaction could relate to oncogenesis. This idea is supported by recent studies reporting up-regulation of APP in several cancers due to its growth-promoting function [65-68]. Mutations that destabilize this interaction may prevent or reduce the degradation of Ab by LRP and cause a rise in APP level [63], which may eventually lead to increased cancer cellular proliferation.

### The structural interaction of A2M with KLK13, and its implication in cancer

**Figure 9:**
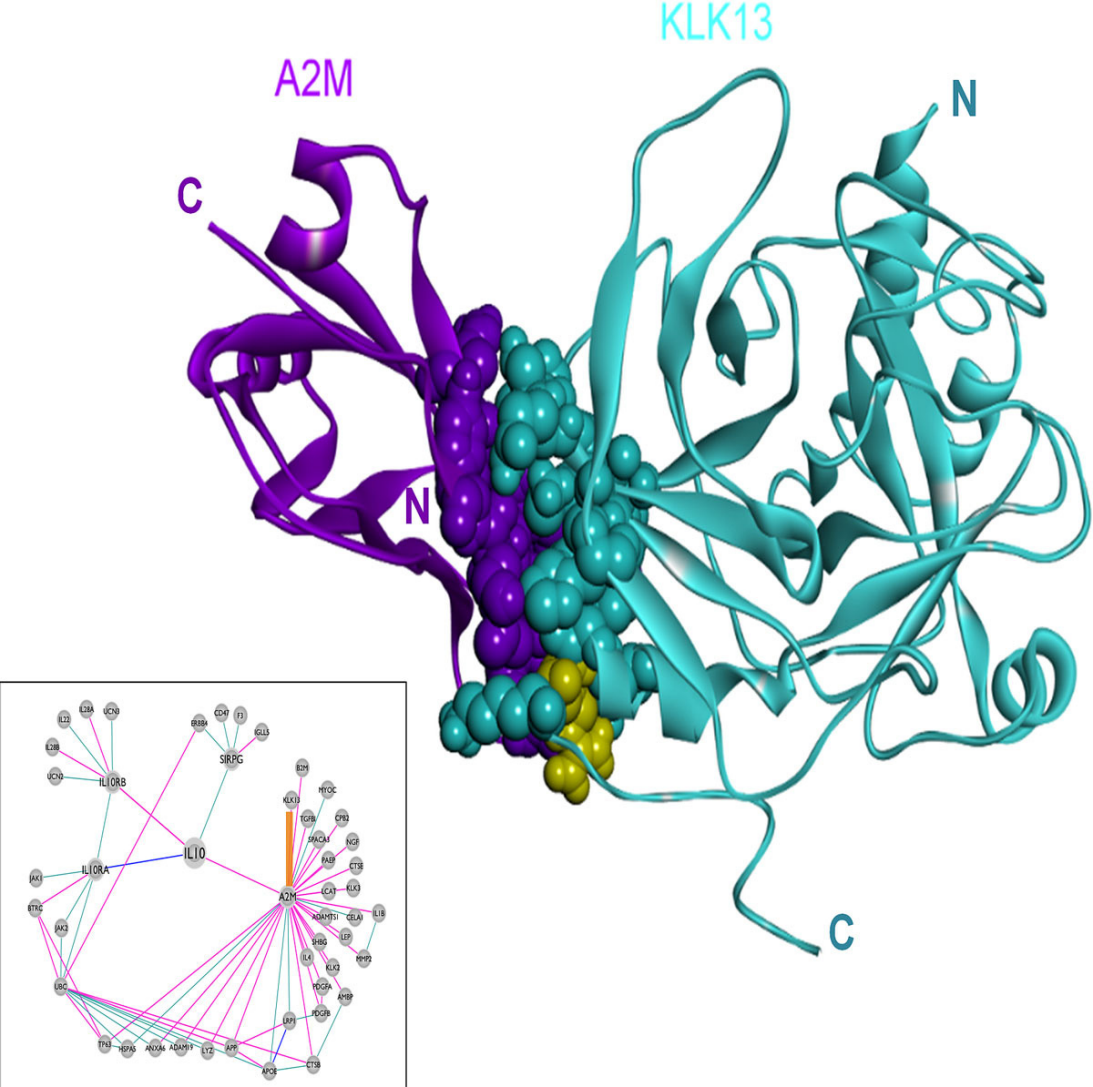
**The predicted complex structure of mutated A2M (1bv8A, purple) and wt KLK13 (PSA, model, blue) with an energy score of -65.22**. Template interface: 1g8tAB, a homodimer interface of nuclease. R263, highlighted in yellow, is the mutated residue.

Kallikrein-13 (KLK13, hK13) is a member of the kallikrein family and encodes a secreted trypsin-like serine protease, which is regulated by steroid hormones [69,70]. Experiments show its ability to cleave the major components of the extracellular matrix [71]. It is known that KLK13 can form a complex with the serine protease inhibitors, including A2M [72]. PRISM was able to predict an interaction between KLK13 and A2M with an energy score of -65.22 (Figure 9 and Table 2). The prediction is between the PDB structure 1bv8A (A2M) and a homology model of KLK13 obtained by the I-TASSER server [47]. The template interface used in the prediction is 1g8tAB, a homodimer interface of nuclease.

Although its function is still unclear, KLK13 is used as a new cancer biomarker in various cancers, including prostate [73], breast [74], ovarian [75], salivary gland [71], testicular [76], and non-small cell lung [77] cancers. This protein may be involved in the promotion of cancer cell growth, angiogenesis, metastasis and invasion. The ramification of the R263L mutation on KLK13 is observed in carcinoma from the COSMIC database [49,50]. We investigated whether this mutation disrupts the interaction of KLK13 - A2M, created a structurally predicted mutant KLK13 protein and minimized its energy. An interaction between the minimized form of wild type KLK13 and A2M is predicted with an energy score of -87.73 (implying that the effect of minimization is insignificant). However, PRISM could not find a favorable interaction between mutant KLK13 and A2M (the best prediction has an energy score of +19.48, Table 2), suggesting disruption of the interaction. Our data is consistent with the prediction from the HotPoint server. Disruption of this interaction can allow KLK13 to react with other proteins, which may lead to cleavage in the major components of the extracellular matrix [71] and help in the promotion of cancer cell growth, metastasis and invasion.

## Conclusions

Structural PPI networks indicate not only which proteins interact, but also how they interact and the location of the interaction sites. Computational techniques allow us to predict PPIs, mutate proteins and investigate the effect of those mutations on the PPIs. Here we constructed the structural PPI network to explore mutational and pathogenesis mechanisms in inflammatory diseases and cancer. We focused on the IL-10 centered network, as IL-10 is a well-known cytokine with an anti-inflammatory activity and relation to cancer. Currently available structural data of the IL-10 pathway are incomplete, with only 2 interactions available in the PDB. We utilized homology modeling to obtain the missing protein structures and a motif-based PPI prediction tool to complete the missing network parts. First we modeled the structures of 10 proteins and then provided models for 40 additional interactions. Although PRISM has a high prediction accuracy, its success to predict interactions is dependent on the conformation of the proteins given. We exploited the structures in the PDB. If the PDB does not include a conformation close to the bound form of the protein, PRISM cannot predict the interaction. That is why we missed some interactions on the network. As the PDB gets richer of structures and different conformations, the success of PRISM to predict interactions will increase. However, the structural PPI network was extended form 2 interactions to 42 interactions via predictions. This allowed us to investigate the effect of clinically observed cancer mutations on our IL-10 centered network. Comparing the interaction models of the wild type and mutant proteins, we observed that specific mutations disrupt the interactions, such as between IL-10 and its receptor, IL-10 and A2M, and A2M and its partners, which may disrupt immune regulation in cancer. We discovered that mutations of the residues, which were clinically observed in cancers as hot spots, change the binding energy and abolished or weakened the interactions. Disruption of the interactions of IL-10 with its receptors (IL-10RA and IL-10RB) and α-2-macroglobulin (A2M) may lead to enhanced inflammation, which could promote tumor growth; blockage of the A2M-APP interaction may lead to cancerous cellular proliferation through free APP; blockage of A2M-KLK13 (hK13) interaction can increase free hK13, which can promote cancer cell growth, metastasis and invasion through damage in the extracellular matrix. Collectively, by merging mutational and structural data - available and predicted using our powerful PRISM tool - and combining it with functional data, we are able to reveal the consequences of weakening or abolishing key interactions, and obtain experimentally-testable mechanisms of oncogenic mutations in the IL-10 network.

## Competing interests

The authors declare that they have no competing interests.

## Authors' contributions

SEAO, HBE, EGM, GK, SM devised the study, carried out the modeling and analysis and wrote the paper; AB wrote the new more efficient and capable version of PRISM used here; ZC edited the manuscript and provided IL-10 and related references; ZC and CVW provided the expertise in the IL-10 protein interactions, the oncogenic mutations, and the relation to inflammation and cancer; AG, OK and RN conceived and oversaw the project, and helped in the writing. These authors contributed equally and appear in alphabetical order: Saliha Ece Acuner-Ozbabacan, Hatice Billur Engin, Emine Guven-Maiorov, Guray Kuzu, and Serena Muratcioglu.

## Supplementary Material

Additional file 1 **The list of proteins in the IL-10 protein-protein interaction network**. The third column provides the degree of contiguity of the proteins to IL-10 protein. For example, if a protein is a first-degree neighbor of IL-10 then its distance from IL-10 is 1. Click here for file

Additional file 3 The list of COSMIC “missense” and “coding silent” mutations mapped onto the target structures in IL-10 centered network Click here for file

Additional file 2 The list of interactions in the IL-10 protein-protein interaction network Click here for file

## Declarations

The publication costs for this article were funded by a grant from NIDCD/NIH Intramural projects Z01-DC-00073 and Z01-DC-00074 and this project has been funded in whole or in part with Federal funds from the National Cancer Institute, National Institutes of Health, under contract number HHSN261200800001E.

This article has been published as part of *BMC Genomics *Volume 15 Supplement 4, 2014: SNP-SIG 2013: Identification and annotation of genetic variants in the context of structure, function, and disease. The full contents of the supplement are available online at http://www.biomedcentral.com/bmcgenomics/supplements/15/S4

## Supplementary Files

**Table S2.**The list of interactions in the IL-10 protein-protein interaction network. 2
