# Supplementary material for: The structural network of Interleukin-10 and its implications in inflammation and cancer
Source: BMC Genomics. 2014 May 20;15(Suppl 4):S2. doi: 10.1186/1471-2164-15-S4-S2 (PMC4083408; doi:10.1186/1471-2164-15-S4-S2)
Supplement: Additional file 1 — The list of proteins in the IL-10 protein-protein interaction network. The third column provides the degree of contiguity of the proteins to IL-10 protein. For example, if a protein is a first-degree neighbor of IL-10 then its distance from IL-10 is 1. [file 1471-2164-15-S4-S2-S1.docx]

Table S1. The list of proteins in the IL-10 protein-protein interaction network. The third column provides the degree of contiguity of the proteins to IL-10 protein. For example, if a protein is a first-degree neighbor of IL-10 then its distance from IL-10 is 1.

| **Protein Abbreviation** | **Protein Name** | **Distance from IL-10** | **Source of Structural Data** |
| --- | --- | --- | --- |
| A2M | Alpha-2-macroglobulin | 1 | PDB |
| ADAM19 | Disintegrin and metalloproteinase domain-containing protein 19 | 2 | Homology Modeling |
| ADAMTS1 | A disintegrin and metalloproteinase with thrombospondin motifs 1 | 2 | PDB |
| AMBP | Alpha-1 microglycoprotein | 2 | PDB |
| ANXA6 | Annexin A6 | 2 | PDB |
| APOE | Apolipoprotein E | 2 | PDB |
| APP | Amyloid beta A4 protein | 2 | PDB |
| B2M | Beta-2-microglobulin | 2 | PDB |
| BTRC | F-box/WD repeat-containing protein 1A | 2 | PDB |
| CD47 | Leukocyte surface antigen CD47 | 2 | PDB |
| CELA1 | Chymotrypsin-like elastase family member 1 | 2 | Homology Modeling |
| CPB2 | Carboxypeptidase B2 | 2 | PDB |
| CTSB | Cathepsin B | 2 | PDB |
| CTSE | Cathepsin E | 2 | PDB |
| ERBB4 | Receptor tyrosine-protein kinase erbB-4 | 2 | PDB |
| F3 | Tissue factor | 2 | PDB |
| HSPA5 | 78 kDa glucose-regulated protein | 2 | PDB |
| IGHV3-6 | Ig heavy chain V region 3-6 | 2 | N/A |
| IGLL5 | Immunoglobulin lambda-like polypeptide 5 | 2 | Homology Modeling |
| IL10 | Interleukin-10 | 0 | PDB |
| IL10RA | Interleukin-10 receptor subunit alpha | 1 | PDB |
| IL10RB | Interleukin-10 receptor subunit beta | 1 | PDB |
| IL1B | Interleukin-1 beta | 2 | PDB |
| IL22 | Interleukin-22 | 2 | PDB |
| IL28A | Interleukin 28A | 2 | Homology Modeling |
| IL28B | Interferon lambda-3 | 2 | PDB |
| IL4 | Interleukin 4 | 2 | PDB |
| JAK1 | Tyrosine-protein kinase JAK1 | 2 | PDB |
| JAK2 | Tyrosine-protein kinase JAK2 | 2 | PDB |
| KLK13 | Kallikrein-13 | 2 | Homology Modeling |
| KLK2 | Kallikrein-2 | 2 | Homology Modeling |
| KLK3 | Prostate-specific antigen | 2 | PDB |
| LCAT | Phosphatidylcholine-sterol acyltransferase | 2 | Homology Modeling |
| LEP | Leptin | 2 | PDB |
| LRP1 | Prolow-density lipoprotein receptor-related protein 1 | 2 | PDB |
| LYZ | Lysozyme | 2 | PDB |
| MMP2 | 72 kDa type IV collagenase | 2 | PDB |
| MYOC | Myocilin | 2 | Homology Modeling |
| NGF | Beta-nerve growth factor | 2 | PDB |
| PAEP | Glycodelin | 2 | Homology Modeling |
| PDGFA | Platelet-derived growth factor subunit A | 2 | PDB |
| PDGFB | Platelet-derived growth factor subunit B | 2 | PDB |
| SHBG | Sex hormone binding globulin | 2 | PDB |
| SIRPG | Signal-regulatory protein gamma | 1 | PDB |
| SPACA3 | Sperm acrosome membrane-associated protein 3 | 2 | Homology Modeling |
| TGFBI | Transforming growth factor-beta-induced protein ig-h3 | 2 | PDB |
| TP63 | Tumor protein 63 | 2 | PDB |
| UBC | Polyubiquitin-C | 2 | PDB |
| UCN2 | Urocortin-2 | 2 | PDB |
| UCN3 | Urocortin-3 | 2 | PDB |
