# Supplementary material for: The structural network of Interleukin-10 and its implications in inflammation and cancer
Source: BMC Genomics. 2014 May 20;15(Suppl 4):S2. doi: 10.1186/1471-2164-15-S4-S2 (PMC4083408; doi:10.1186/1471-2164-15-S4-S2)
Supplement: Additional file 3 — The list of COSMIC “missense” and “coding silent” mutations mapped onto the target structures in IL-10 centered network [file 1471-2164-15-S4-S2-S3.docx]

**Table S3.** The list of COSMIC “missense” and “coding silent” mutations mapped onto the target structures in IL-10 centered network

| Protein | PDB | Mutation | Protein | PDB | Mutation | Protein | PDB | Mutation | Protein | PDB | Mutation |
| --- | --- | --- | --- | --- | --- | --- | --- | --- | --- | --- | --- |
| A2M | 1bv8A | L1348L | ANXA6 | 1m9iA | P277Q | CPB2 | 3d68A | G368V | IL1B | 3ltqA | I222I |
| A2M | 1bv8A | D1353E | ANXA6 | 1m9iA | L295L | CPB2 | 3d68A | D375E | IL1B | 3ltqA | M246I |
| A2M | 1bv8A | P1355H | ANXA6 | 1m9iA | M330I | CPB2 | 3d68A | F409S | IL1B | 3ltqA | G256G |
| A2M | 1bv8A | S1360R | ANXA6 | 1m9iA | R333C | CPB2 | 3d68A | K414Q | IL1B | 3ltqA | F262L |
| A2M | 1bv8A | F1389F | ANXA6 | 1m9iA | Y354C | CTSB | 3pbhA | R18Q | IL28B | 3hhcB | C69W |
| A2M | 1bv8A | T1395K | ANXA6 | 1m9iA | K366N | CTSB | 3pbhA | P25P | IL28B | 3hhcB | R72C |
| A2M | 1bv8A | Y1463D | ANXA6 | 1m9iA | R410H | CTSB | 3pbhA | Y33H | IL28B | 3hhcB | R78R |
| A2M | 2p9rA | V129V | ANXA6 | 1m9iA | R445C | CTSB | 3pbhA | N38T | IL28B | 3hhcB | R90H |
| A2M | 2p9rA | V144L | ANXA6 | 1m9iA | Q451R | CTSB | 3pbhA | C59F | IL28B | 3hhcB | L94F |
| A2M | 2p9rA | F146V | ANXA6 | 1m9iA | A478V | CTSB | 3pbhA | I99M | IL28B | 3hhcB | L100L |
| A2M | 2p9rA | R147C | ANXA6 | 1m9iA | R479K | CTSB | 3pbhA | C105R | IL28B | 3hhcB | T101M |
| A2M | 2p9rA | V148V | ANXA6 | 1m9iA | E538D | CTSB | 3pbhA | T140T | IL28B | 3hhcB | L102P |
| A2M | 2p9rA | S150S | ANXA6 | 1m9iA | R551K | CTSB | 3pbhA | G143D | IL28B | 3hhcB | K103E |
| A2M | 2p9rA | N159Y | ANXA6 | 1m9iA | S555Y | CTSB | 3pbhA | C179Y | IL28B | 3hhcB | H124N |
| A2M | 2p9rA | P163S | ANXA6 | 1m9iA | T558T | CTSB | 3pbhA | I184V | IL28B | 3hhcB | S131S |
| A2M | 2p9rA | R174C | ANXA6 | 1m9iA | T623I | CTSB | 3pbhA | V191I | IL28B | 3hhcB | L133F |
| A2M | 2p9rA | I175I | ANXA6 | 1m9iA | V632I | CTSB | 3pbhA | S194C | IL28B | 3hhcB | I137T |
| A2M | 2p9rA | A176T | ANXA6 | 1m9iA | A651T | CTSB | 3pbhA | P248P | IL28B | 3hhcB | R146S |
| A2M | 2p9rA | E184D | ANXA6 | 1m9iA | D652E | CTSB | 3pbhA | A252V | IL28B | 3hhcB | R150C |
| A2M | 2p9rA | S191Y | ANXA6 | 1m9iA | K653T | CTSB | 3pbhA | S257L | IL28B | 3hhcB | F176F |
| A2M | 2p9rA | P193S | ANXA6 | 1m9iA | M658T | CTSB | 3pbhA | H269Q | IL28B | 3hhcB | L178L |
| A2M | 2p9rA | P218H | ANXA6 | 1m9iA | R670T | CTSB | 3pbhA | A279T | IL28B | 3hhcB | R180H |
| A2M | 2p9rA | T220T | ANXA6 | 1m9iA | M672I | CTSB | 3pbhA | R281H | IL28B | 3hhcB | T183M |
| A2M | 4acqC | P29Q | APOE | 2kc3A | R43L | CTSB | 3pbhA | T291T | IL28B | 3hhcB | S191S |
| A2M | 4acqC | D82D | APOE | 2kc3A | A47T | CTSB | 3pbhA | P292P | IL28B | 3hhcB | G192R |
| A2M | 4acqC | A88T | APOE | 2kc3A | R56C | CTSB | 3pbhA | E322K | IL4 | 1bbnA | E50K |
| A2M | 4acqC | A90T | APOE | 2kc3A | A109A | ERBB4 | 2ahxB | L39F | IL4 | 1bbnA | H83Y |
| A2M | 4acqC | P92T | APOE | 2kc3A | S147N | ERBB4 | 2ahxB | R47G | IL4 | 1bbnA | R88C |
| A2M | 4acqC | M121I | APOE | 2l7bA | R43L | ERBB4 | 2ahxB | R50H | IL4 | 1bbnA | A92V |
| A2M | 4acqC | N124N | APOE | 2l7bA | A47T | ERBB4 | 2ahxB | C56Y | IL4 | 1bbnA | K101R |
| A2M | 4acqC | V129V | APOE | 2l7bA | R56C | ERBB4 | 2ahxB | V59D | IL4 | 1bbnA | A118V |
| A2M | 4acqC | V144L | APOE | 2l7bA | A109A | ERBB4 | 2ahxB | E69K | IL4 | 1bbnA | T132M |
| A2M | 4acqC | F146V | APOE | 2l7bA | S147N | ERBB4 | 2ahxB | D73V | IL4 | 1bbnA | F136C |
| A2M | 4acqC | R147C | APOE | 2l7bA | R258H | ERBB4 | 2ahxB | S75F | IL4 | 1bbnA | S152L |
| A2M | 4acqC | V148V | APOE | 2l7bA | R278L | ERBB4 | 2ahxB | S79F | KLK3 | 2zchP | V26V |
| A2M | 4acqC | S150S | APOE | 2l7bA | A304E | ERBB4 | 2ahxB | V89A | KLK3 | 2zchP | C31C |
| A2M | 4acqC | N159Y | APP | 1owtA | A126V | ERBB4 | 2ahxB | A90T | KLK3 | 2zchP | E32G |
| A2M | 4acqC | P163S | APP | 1owtA | C144C | ERBB4 | 2ahxB | R103C | KLK3 | 2zchP | A43V |
| A2M | 4acqC | R174C | APP | 1owtA | E145K | ERBB4 | 2ahxB | R106C | KLK3 | 2zchP | R45H |
| A2M | 4acqC | I175I | APP | 1owtA | A154T | ERBB4 | 2ahxB | Y111H | KLK3 | 2zchP | C50C |
| A2M | 4acqC | A176T | APP | 1owtA | C158Y | ERBB4 | 2ahxB | R114Q | KLK3 | 2zchP | V55M |
| A2M | 4acqC | E184D | APP | 1owtA | E183Q | ERBB4 | 2ahxB | L117L | KLK3 | 2zchP | L61L |
| A2M | 4acqC | S191Y | APP | 1tknA | R469H | ERBB4 | 2ahxB | A118S | KLK3 | 2zchP | I73I |
| A2M | 4acqC | P193S | APP | 1tknA | P484S | ERBB4 | 2ahxB | G130E | KLK3 | 2zchP | R77G |
| A2M | 4acqC | P218H | APP | 1tknA | R488C | ERBB4 | 2ahxB | T140A | KLK3 | 2zchP | H78Q |
| A2M | 4acqC | T220T | APP | 1tknA | F491L | ERBB4 | 2ahxB | N152K | KLK3 | 2zchP | L80L |
| A2M | 4acqC | F229S | APP | 1tknA | N492S | ERBB4 | 2ahxB | C156R | KLK3 | 2zchP | L100L |
| A2M | 4acqC | Y254Y | APP | 1tknA | V498I | ERBB4 | 2ahxB | I166N | KLK3 | 2zchP | D102E |
| A2M | 4acqC | R270K | APP | 1tknA | R499C | ERBB4 | 2ahxB | R168W | KLK3 | 2zchP | L105L |
| A2M | 4acqC | K271N | APP | 1tknA | F512F | ERBB4 | 2ahxB | P172R | KLK3 | 2zchP | G149W |
| A2M | 4acqC | D274D | APP | 1tknA | E513K | ERBB4 | 2ahxB | T180A | KLK3 | 2zchP | T150T |
| A2M | 4acqC | A275D | APP | 1tknA | V515V | ERBB4 | 2ahxB | N181D | KLK3 | 2zchP | Y153Y |
| A2M | 4acqC | G280G | APP | 1tknA | M541I | ERBB4 | 2ahxB | G187G | KLK3 | 2zchP | A154D |
| A2M | 4acqC | Q284H | APP | 1tknA | E556K | ERBB4 | 2ahxB | H190R | KLK3 | 2zchP | G156V |
| A2M | 4acqC | K289T | APP | 1tknA | E564Q | ERBB4 | 2ahxB | T201K | KLK3 | 2zchP | V174V |
| A2M | 4acqC | N295Y | APP | 2llmA | A701V | ERBB4 | 2ahxB | H204H | KLK3 | 2zchP | D182A |
| A2M | 4acqC | T306N | APP | 2llmA | G708G | ERBB4 | 2ahxB | T207S | KLK3 | 2zchP | F195F |
| A2M | 4acqC | L311P | APP | 2llmA | G709A | ERBB4 | 2ahxB | T211M | KLK3 | 2zchP | R201R |
| A2M | 4acqC | H358Q | APP | 2llmA | T714A | ERBB4 | 2ahxB | C217R | KLK3 | 2zchP | C209Y |
| A2M | 4acqC | R360R | APP | 2llmA | I716I | ERBB4 | 2ahxB | V226I | KLK3 | 2zchP | G215G |
| A2M | 4acqC | F365L | APP | 3ktmE | L28P | ERBB4 | 2ahxB | C234Y | KLK3 | 2zchP | G230D |
| A2M | 4acqC | G367R | APP | 3ktmE | G68D | ERBB4 | 2ahxB | A235V | KLK3 | 2zchP | A235V |
| A2M | 4acqC | P380Q | APP | 3ktmE | A126V | ERBB4 | 2ahxB | T244A | KLK3 | 2zchP | E238K |
| A2M | 4acqC | S395F | APP | 3ktmE | C144C | ERBB4 | 2ahxB | M250I | LEP | 1ax8A | N93N |
| A2M | 4acqC | R422R | APP | 3ktmE | E145K | ERBB4 | 2ahxB | F252L | LEP | 1ax8A | L101L |
| A2M | 4acqC | D427N | APP | 3ktmE | A154T | ERBB4 | 2ahxB | T265A | LEP | 1ax8A | R105R |
| A2M | 4acqC | E442K | APP | 3ktmE | C158Y | ERBB4 | 2ahxB | Y268C | LEP | 1ax8A | H109L |
| A2M | 4acqC | F452L | APP | 3ktmE | E183Q | ERBB4 | 2ahxB | H277N | LEP | 1ax8A | L128V |
| A2M | 4acqC | S453C | APP | 3ktmE | E191Q | ERBB4 | 2ahxB | Y285C | LEP | 1ax8A | S130N |
| A2M | 4acqC | L461L | APP | 3nylA | D376G | ERBB4 | 2ahxB | C293F | LEP | 1ax8A | V134V |
| A2M | 4acqC | V476V | APP | 3nylA | K393E | ERBB4 | 2ahxB | H295Y | LEP | 1ax8A | E136D |
| A2M | 4acqC | P529H | APP | 3nylA | E399Q | ERBB4 | 2ahxB | S302G | LEP | 1ax8A | V144V |
| A2M | 4acqC | T548I | APP | 3nylA | R418C | ERBB4 | 2ahxB | S303Y | LEP | 1ax8A | L158L |
| A2M | 4acqC | L578L | APP | 3nylA | K421N | ERBB4 | 2ahxB | C304F | LEP | 1ax8A | G166V |
| A2M | 4acqC | V594V | APP | 3nylA | K428E | ERBB4 | 2ahxB | R306S | LRP1 | 2knyA | P2776L |
| A2M | 4acqC | R598C | APP | 3nylA | V439V | ERBB4 | 2ahxB | M313I | LRP1 | 2knyA | R2791C |
| A2M | 4acqC | S617L | APP | 3nylA | S441Y | ERBB4 | 2ahxB | E317K | LRP1 | 2knyA | C2794Y |
| A2M | 4acqC | L621P | APP | 3nylA | R469H | ERBB4 | 2ahxB | G319E | LRP1 | 2knyA | C2800F |
| A2M | 4acqC | K625R | APP | 3nylA | P484S | ERBB4 | 2ahxB | I320F | LRP1 | 2knyA | C2825L |
| A2M | 4acqC | D638E | APP | 3nylA | R488C | ERBB4 | 2ahxB | K321N | LRP1 | 2knyA | R2828C |
| A2M | 4acqC | S660R | APP | 3nylA | F491L | ERBB4 | 2ahxB | T327I | LRP1 | 2knyA | D2845H |
| A2M | 4acqC | K664N | APP | 3nylA | N492S | ERBB4 | 2ahxB | D335Y | LYZ | 1lz6A | R28I |
| A2M | 4acqC | F669V | APP | 3nylA | V498I | ERBB4 | 2ahxB | S341L | LYZ | 1lz6A | R59L |
| A2M | 4acqC | T730S | APP | 3nylA | R499C | ERBB4 | 2ahxB | V348M | LYZ | 1lz6A | R119C |
| A2M | 4acqC | R732Q | APP | 3nylA | F512F | ERBB4 | 2ahxB | N352S | LYZ | 1lz6A | R131I |
| A2M | 4acqC | E737E | APP | 3nylA | E513K | ERBB4 | 2ahxB | I353N | LYZ | 1lz6A | R133H |
| A2M | 4acqC | V758V | APP | 3nylA | V515V | ERBB4 | 2ahxB | N363N | LYZ | 1lz6A | R140S |
| A2M | 4acqC | P759T | APP | 3nylA | M541I | ERBB4 | 2ahxB | G375G | MMP2 | 3ayuA | P117T |
| A2M | 4acqC | L772M | APP | 3nylA | E556K | ERBB4 | 2ahxB | P386Q | MMP2 | 3ayuA | I128I |
| A2M | 4acqC | G779D | APP | 3nylA | E564Q | ERBB4 | 2ahxB | E387Q | MMP2 | 3ayuA | A145V |
| A2M | 4acqC | S782C | APP | 3umkA | K393E | ERBB4 | 2ahxB | N390I | MMP2 | 3ayuA | S152S |
| A2M | 4acqC | S785P | APP | 3umkA | E399Q | ERBB4 | 2ahxB | R393W | MMP2 | 3ayuA | P156P |
| A2M | 4acqC | P799L | APP | 3umkA | R418C | ERBB4 | 2ahxB | T394T | MMP2 | 3ayuA | R158Q |
| A2M | 4acqC | T813T | APP | 3umkA | K421N | ERBB4 | 2ahxB | P409L | MMP2 | 3ayuA | F159C |
| A2M | 4acqC | L835I | APP | 3umkA | K428E | ERBB4 | 2ahxB | L420M | MMP2 | 3ayuA | R161Q |
| A2M | 4acqC | I848I | APP | 3umkA | V439V | ERBB4 | 2ahxB | L428L | MMP2 | 3ayuA | G186R |
| A2M | 4acqC | N851N | APP | 3umkA | S441Y | ERBB4 | 2ahxB | Y429Y | MMP2 | 3ayuA | G189R |
| A2M | 4acqC | G852R | APP | 3umkA | R469H | ERBB4 | 2ahxB | L432M | MMP2 | 3ayuA | P197T |
| A2M | 4acqC | R853R | APP | 3umkA | P484S | ERBB4 | 2ahxB | L445L | MMP2 | 3ayuA | G202G |
| A2M | 4acqC | V860A | APP | 3umkA | R488C | ERBB4 | 2ahxB | Q448H | MMP2 | 3ayuA | D209D |
| A2M | 4acqC | T871N | APP | 3umkA | F491L | ERBB4 | 2ahxB | L450P | MMP2 | 3ayuA | L215L |
| A2M | 4acqC | S873S | APP | 3umkA | N492S | ERBB4 | 2ahxB | E452K | MMP2 | 3ayuA | V400L |
| A2M | 4acqC | A874T | APP | 3umkA | V498I | ERBB4 | 2ahxB | Y459N | MMP2 | 3ayuA | H407Y |
| A2M | 4acqC | G884G | APP | 3umkA | R499C | ERBB4 | 2ahxB | T461A | MMP2 | 3ayuA | P417S |
| A2M | 4acqC | T885A | APP | 3umkA | F512F | ERBB4 | 2ahxB | S464G | MMP2 | 3ayuA | A422E |
| A2M | 4acqC | L903L | APP | 3umkA | E513K | ERBB4 | 2ahxB | Y468F | MMP2 | 3ayuA | Y425N |
| A2M | 4acqC | R945Q | APP | 3umkA | V515V | ERBB4 | 2ahxB | T475P | MMP2 | 3ayuA | R432S |
| A2M | 4acqC | D953G | APP | 3umkA | M541I | ERBB4 | 2ahxB | L477P | NGF | 1wwwW | S134S |
| A2M | 4acqC | L965R | APP | 3umkA | E556K | ERBB4 | 2ahxB | F478V | NGF | 1wwwW | S140S |
| A2M | 4acqC | P969S | APP | 3umkA | E564Q | ERBB4 | 2ahxB | T480T | NGF | 1wwwW | V141M |
| A2M | 4acqC | V978V | B2M | 1ypzB | K26E | ERBB4 | 2ahxB | Q483E | NGF | 1wwwW | A149T |
| A2M | 4acqC | S1002Y | B2M | 1ypzB | Y30H | ERBB4 | 2ahxB | R488W | NGF | 1wwwW | K155K |
| A2M | 4acqC | L1008L | B2M | 1ypzB | E36E | ERBB4 | 2ahxB | D489E | NGF | 1wwwW | C189C |
| A2M | 4acqC | S1026N | B2M | 1ypzB | L43P | ERBB4 | 2ahxB | R491K | NGF | 1wwwW | R190G |
| A2M | 4acqC | G1029V | B2M | 1ypzB | N44I | ERBB4 | 2ahxB | G500E | NGF | 1wwwW | K195K |
| A2M | 4acqC | R1034S | B2M | 1ypzB | C45G | ERBB4 | 2ahxB | W513R | NGF | 1wwwW | A210V |
| A2M | 4acqC | A1043T | B2M | 1ypzB | V47M | ERBB4 | 2ahxB | D518G | NGF | 1wwwW | R224Q |
| A2M | 4acqC | Q1051H | B2M | 1ypzB | S53S | ERBB4 | 2ahxB | L521M | PDGFA | 3mjkA | S58Y |
| A2M | 4acqC | Y1055C | B2M | 1ypzB | D54N | ERBB4 | 2ahxB | R530S | PDGFA | 3mjkA | S62S |
| A2M | 4acqC | I1056I | B2M | 1ypzB | N62T | ERBB4 | 2ahxB | E534Q | PDGFA | 3mjkA | L63V |
| A2M | 4acqC | A1061V | B2M | 1ypzB | I66N | ERBB4 | 2ahxB | S535T | PDGFA | 3mjkA | K72N |
| A2M | 4acqC | T1064A | B2M | 1ypzB | H71N | ERBB4 | 2ahxB | D540N | PDGFA | 3mjkA | E76K |
| A2M | 4acqC | L1067I | B2M | 1ypzB | F76C | ERBB4 | 2ahxB | E542K | PDGFA | 3mjkA | P93P |
| A2M | 4acqC | R1073R | B2M | 1ypzB | F82V | ERBB4 | 2ahxB | R544W | SHBG | 1kdkA | E106Q |
| A2M | 4acqC | Q1074K | B2M | 1ypzB | Y83F | ERBB4 | 2ahxB | N548T | SHBG | 1kdkA | A114T |
| A2M | 4acqC | S1083Y | B2M | 1ypzB | L84R | ERBB4 | 2ahxB | C552C | SHBG | 1kdkA | P159L |
| A2M | 4acqC | I1091T | B2M | 1ypzB | Y86N | ERBB4 | 2ahxB | V553V | SHBG | 1kdkA | A171A |
| A2M | 4acqC | S1102C | B2M | 1ypzB | E94K | ERBB4 | 2ahxB | E563K | SHBG | 1kdkA | L172I |
| A2M | 4acqC | T1106T | B2M | 1ypzB | E97K | ERBB4 | 2ahxB | G565G | SHBG | 1kdkA | R195H |
| A2M | 4acqC | P1119P | B2M | 1ypzB | Y98N | ERBB4 | 2ahxB | L567F | SIRPG | 2jjwA | G46R |
| A2M | 4acqC | E1129Q | B2M | 1ypzB | V113G | ERBB4 | 2ahxB | P574T | SIRPG | 2jjwA | S57F |
| A2M | 4acqC | E1137Q | B2M | 1ypzB | M119L | ERBB4 | 2ahxB | C580C | SIRPG | 2jjwA | P60P |
| A2M | 4acqC | D1139E | B2M | 3ov6A | M1L | ERBB4 | 2ahxB | H582Y | SIRPG | 2jjwA | G62E |
| A2M | 4acqC | A1151P | B2M | 3ov6A | R3C | ERBB4 | 2ahxB | F583Y | SIRPG | 2jjwA | R74Q |
| A2M | 4acqC | K1176M | B2M | 3ov6A | A6V | ERBB4 | 2ahxB | G586D | SIRPG | 2jjwA | Q80H |
| A2M | 4acqC | A1201T | B2M | 3ov6A | L7S | ERBB4 | 2ahxB | C589F | SIRPG | 2jjwA | D101Y |
| A2M | 4acqC | P1202H | B2M | 3ov6A | A8T | ERBB4 | 2ahxB | D595G | SIRPG | 2jjwA | R105C |
| A2M | 4acqC | A1204V | B2M | 3ov6A | L12Q | ERBB4 | 2ahxB | Q598L | SIRPG | 2jjwA | S108S |
| A2M | 4acqC | T1209A | B2M | 3ov6A | L13F | ERBB4 | 2ahxB | D609N | SIRPG | 2jjwA | R123Q |
| A2M | 4acqC | Y1216Y | B2M | 3ov6A | L15F | ERBB4 | 2ahxB | P610T | SIRPG | 2jjwA | M140I |
| A2M | 4acqC | T1229S | B2M | 3ov6A | Y30H | ERBB4 | 2ahxB | R612Q | SIRPG | 2jjwA | A141S |
| A2M | 4acqC | T1239T | B2M | 3ov6A | E36E | ERBB4 | 2ahxB | H618Y | TGFBI | 1x3bA | M502V |
| A2M | 4acqC | K1240N | B2M | 3ov6A | L43P | ERBB4 | 2ahxB | P619L | TGFBI | 1x3bA | T504N |
| A2M | 4acqC | F1248L | B2M | 3ov6A | N44I | ERBB4 | 2ahxB | G624G | TGFBI | 1x3bA | V505F |
| A2M | 4acqC | Q1252H | B2M | 3ov6A | C45G | ERBB4 | 2ahxB | P628H | TGFBI | 1x3bA | D507V |
| A2M | 4acqC | Q1281K | B2M | 3ov6A | V47M | ERBB4 | 2ahxB | P637S | TGFBI | 1x3bA | L509P |
| A2M | 4acqC | T1285I | B2M | 3ov6A | S53S | ERBB4 | 2ahxB | W638L | TGFBI | 1x3bA | I522I |
| A2M | 4acqC | F1290L | B2M | 3ov6A | D54N | IL10 | 2ilkA | T24T | TGFBI | 1x3bA | E529K |
| A2M | 4acqC | R1297H | B2M | 3ov6A | N62T | IL10 | 2ilkA | N63S | TGFBI | 1x3bA | R533R |
| A2M | 4acqC | P1306Q | B2M | 3ov6A | I66N | IL10 | 2ilkA | C80Y | TGFBI | 1x3bA | E576K |
| A2M | 4acqC | G1310W | B2M | 3ov6A | H71N | IL10 | 2ilkA | A107V | TGFBI | 1x3bA | K590T |
| A2M | 4acqC | M1314I | B2M | 3ov6A | F76C | IL10 | 2ilkA | V109V | TGFBI | 1x3bA | G594V |
| A2M | 4acqC | G1318R | B2M | 3ov6A | F82V | IL10 | 2ilkA | G113R | TGFBI | 1x3bA | N609S |
| A2M | 4acqC | C1321Y | B2M | 3ov6A | Y83F | IL10 | 2ilkA | E114D | TGFBI | 1x3bA | E611K |
| A2M | 4acqC | L1324I | B2M | 3ov6A | L84R | IL10 | 2ilkA | R120R | TGFBI | 1x3bA | P612S |
| A2M | 4acqC | L1348L | B2M | 3ov6A | Y86N | IL10 | 2ilkA | R125C | TGFBI | 1x3bA | N622N |
| A2M | 4acqC | D1353E | B2M | 3ov6A | E94K | IL10 | 2ilkA | C126R | TP63 | 2rmnA | F156F |
| A2M | 4acqC | P1355H | B2M | 3ov6A | E97K | IL10 | 2ilkA | V139M | TP63 | 2rmnA | D157Y |
| A2M | 4acqC | S1360R | B2M | 3ov6A | Y98N | IL10 | 2ilkA | K148K | TP63 | 2rmnA | D170E |
| A2M | 4acqC | F1389F | B2M | 3ov6A | V113G | IL10RA | 1lqsR | T24T | TP63 | 2rmnA | P172P |
| A2M | 4acqC | T1395K | B2M | 3ov6A | M119L | IL10RA | 1lqsR | P30L | TP63 | 2rmnA | P174Q |
| A2M | 4acqC | Y1463D | BTRC | 1p22A | P185P | IL10RA | 1lqsR | N63S | TP63 | 2rmnA | F177F |
| ADAMTS1 | 2jihB | R258H | BTRC | 1p22A | R187W | IL10RA | 1lqsR | C80Y | TP63 | 2rmnA | S184W |
| ADAMTS1 | 2jihB | S269L | BTRC | 1p22A | E214G | IL10RA | 1lqsR | R97W | TP63 | 2rmnA | A190P |
| ADAMTS1 | 2jihB | H274H | BTRC | 1p22A | E230K | IL10RA | 1lqsR | R101Q | TP63 | 2rmnA | I213F |
| ADAMTS1 | 2jihB | L278L | BTRC | 1p22A | M232V | IL10RA | 1lqsR | A107V | TP63 | 2rmnA | R226H |
| ADAMTS1 | 2jihB | S287L | BTRC | 1p22A | S237Y | IL10RA | 1lqsR | V109V | TP63 | 2rmnA | H236R |
| ADAMTS1 | 2jihB | A290E | BTRC | 1p22A | G247V | IL10RA | 1lqsR | G113R | TP63 | 2rmnA | C244Y |
| ADAMTS1 | 2jihB | L304M | BTRC | 1p22A | S266Y | IL10RA | 1lqsR | E114D | TP63 | 2rmnA | R251H |
| ADAMTS1 | 2jihB | K308T | BTRC | 1p22A | A270A | IL10RA | 1lqsR | R120R | TP63 | 2rmnA | N254N |
| ADAMTS1 | 2jihB | L310M | BTRC | 1p22A | I275S | IL10RA | 1lqsR | R125C | TP63 | 2rmnA | G269E |
| ADAMTS1 | 2jihB | E315A | BTRC | 1p22A | S284Y | IL10RA | 1lqsR | C126R | TP63 | 2rmnA | E328G |
| ADAMTS1 | 2jihB | R330W | BTRC | 1p22A | E301Q | IL10RA | 1lqsR | G128D | TP63 | 2rmnA | T329P |
| ADAMTS1 | 2jihB | E348E | BTRC | 1p22A | Y307F | IL10RA | 1lqsR | E133K | TP63 | 2rmnA | R337Q |
| ADAMTS1 | 2jihB | T352T | BTRC | 1p22A | D312N | IL10RA | 1lqsR | V139M | TP63 | 2rmnA | R338H |
| ADAMTS1 | 2jihB | L355I | BTRC | 1p22A | I316T | IL10RA | 1lqsR | K148K | TP63 | 2rmnA | C339F |
| ADAMTS1 | 2jihB | T377N | BTRC | 1p22A | R321Q | IL10RA | 1lqsR | A151T | TP63 | 2rmnA | R343Q |
| ADAMTS1 | 2jihB | P381T | BTRC | 1p22A | L339F | IL10RA | 1lqsR | R165Q | TP63 | 2rmnA | K361K |
| ADAMTS1 | 2jihB | S386F | BTRC | 1p22A | T398T | IL10RA | 1lqsR | Y167H | TP63 | 2rmnA | V364I |
| ADAMTS1 | 2jihB | V387I | BTRC | 1p22A | I416M | IL10RA | 1lqsR | E168D | TP63 | 2rmnA | S365L |
| ADAMTS1 | 2jihB | S427Y | BTRC | 1p22A | R419Q | IL10RA | 1lqsR | T179T | TP63 | 2rmnA | K369N |
| ADAMTS1 | 2jihB | L437L | BTRC | 1p22A | Y438C | IL10RA | 1lqsR | N189K | TP63 | 2rmnA | N370N |
| ADAMTS1 | 2jihB | S440R | BTRC | 1p22A | T455A | IL10RA | 1lqsR | W218C | TP63 | 2rmnA | R379C |
| ADAMTS1 | 2jihB | Q441Q | BTRC | 1p22A | F458L | IL10RA | 1lqsR | C223C | TP63 | 2rmnA | M387I |
| ADAMTS1 | 2jihB | L463F | BTRC | 1p22A | V480V | IL10RB | 3lqmA | T24T | TP63 | 2rmnA | T388I |
| ADAMTS1 | 2jihB | P470L | BTRC | 1p22A | A497A | IL10RB | 3lqmA | F34L | TP63 | 2y9tA | S551G |
| ADAMTS1 | 2jihB | P474T | BTRC | 1p22A | F514Y | IL10RB | 3lqmA | Y56Y | TP63 | 2y9tA | L553L |
| ADAMTS1 | 2jihB | Y482Y | BTRC | 1p22A | G522G | IL10RB | 3lqmA | N63S | TP63 | 2y9tA | T566M |
| ADAMTS1 | 2jihB | R486W | BTRC | 1p22A | T545I | IL10RB | 3lqmA | T70I | TP63 | 2y9tA | T571T |
| ADAMTS1 | 2jihB | V520V | BTRC | 1p22A | D564V | IL10RB | 3lqmA | L79V | TP63 | 2y9tA | E577Q |
| ADAMTS1 | 2jihB | H525Y | BTRC | 1p22A | E565G | IL10RB | 3lqmA | C80Y | TP63 | 2y9tA | D583V |
| ADAMTS1 | 2jihB | P527A | CPB2 | 3d68A | L53L | IL10RB | 3lqmA | R90R | TP63 | 2y9tA | A596V |
| ADAMTS1 | 2jihB | W528S | CPB2 | 3d68A | P56L | IL10RB | 3lqmA | E92K | TP63 | 2y9tA | E609K |
| ADAMTS1 | 2jihB | N542N | CPB2 | 3d68A | V77V | IL10RB | 3lqmA | A107V | TP63 | 4a9zC | E409D |
| ADAMTS1 | 2jihB | D550N | CPB2 | 3d68A | D78N | IL10RB | 3lqmA | V109V | TP63 | 4a9zC | K418R |
| ADAMTS1 | 2jihB | R551I | CPB2 | 3d68A | S87S | IL10RB | 3lqmA | G113R | TP63 | 4a9zC | E419E |
| AMBP | 4es7A | R39P | CPB2 | 3d68A | G88E | IL10RB | 3lqmA | E114D | TP63 | 4a9zC | Q437K |
| AMBP | 4es7A | I40I | CPB2 | 3d68A | R114L | IL10RB | 3lqmA | R120R | TP63 | 4a9zC | Q439Q |
| AMBP | 4es7A | K57N | CPB2 | 3d68A | H148N | IL10RB | 3lqmA | R125C | UBC | 3b0aD | I3I |
| AMBP | 4es7A | V69M | CPB2 | 3d68A | S164F | IL10RB | 3lqmA | C126R | UBC | 3b0aD | S20S |
| AMBP | 4es7A | E72G | CPB2 | 3d68A | A170S | IL10RB | 3lqmA | R130C | UBC | 3b0aD | D21N |
| AMBP | 4es7A | K88T | CPB2 | 3d68A | R183T | IL10RB | 3lqmA | F131L | UBC | 3b0aD | I30V |
| AMBP | 4es7A | W114L | CPB2 | 3d68A | G196C | IL10RB | 3lqmA | V139M | UBC | 3b0aD | G35G |
| AMBP | 4es7A | I116V | CPB2 | 3d68A | F201L | IL10RB | 3lqmA | Y140H | UBC | 3b0aD | R42R |
| AMBP | 4es7A | K136K | CPB2 | 3d68A | V222M | IL10RB | 3lqmA | T144T | UBC | 3b0aD | D52N |
| AMBP | 4es7A | I146M | CPB2 | 3d68A | K233R | IL10RB | 3lqmA | K148K | UBC | 3b0aD | H68H |
| AMBP | 4es7A | Y151H | CPB2 | 3d68A | W238R | IL10RB | 3lqmA | D197H | UBC | 3b0aD | G76G |
| AMBP | 4es7A | E159D | CPB2 | 3d68A | I251I | IL10RB | 3lqmA | R198W | UBC | 3b0aD | V81V |
| ANXA6 | 1m9iA | R63W | CPB2 | 3d68A | D254Y | IL1B | 3ltqA | T125M | UBC | 3b0aD | T98T |
| ANXA6 | 1m9iA | A80T | CPB2 | 3d68A | A260D | IL1B | 3ltqA | P139L | UBC | 3b0aD | I99I |
| ANXA6 | 1m9iA | T83A | CPB2 | 3d68A | E276K | IL1B | 3ltqA | L145L | UBC | 3b0aD | E110K |
| ANXA6 | 1m9iA | S116S | CPB2 | 3d68A | Y282C | IL1B | 3ltqA | V156V | UBC | 3b0aD | A122V |
| ANXA6 | 1m9iA | G140C | CPB2 | 3d68A | P287T | IL1B | 3ltqA | G165E | UBC | 3b0aD | G123A |
| ANXA6 | 1m9iA | A150V | CPB2 | 3d68A | L296F | IL1B | 3ltqA | E180K | UBC | 3b0aD | K124K |
| ANXA6 | 1m9iA | G200G | CPB2 | 3d68A | Q314E | IL1B | 3ltqA | K181N | UBC | 3b0aD | Q125E |
| ANXA6 | 1m9iA | L214R | CPB2 | 3d68A | R324Q | IL1B | 3ltqA | V188M | UBC | 3b0aD | R130C |
| ANXA6 | 1m9iA | S225S | CPB2 | 3d68A | S325R | IL1B | 3ltqA | P194T | UBC | 3b0aD | I137M |
| ANXA6 | 1m9iA | T246A | CPB2 | 3d68A | T351T | IL1B | 3ltqA | P203H | UBC | 3b0aD | Q138R |
| ANXA6 | 1m9iA | I254I | CPB2 | 3d68A | G356D | IL1B | 3ltqA | K204N | UBC | 3b0aD | V146V |
| ANXA6 | 1m9iA | R258C | CPB2 | 3d68A | P366A | IL1B | 3ltqA | K210K |  |  |  |
