# Supplementary material for: The structural network of Interleukin-10 and its implications in inflammation and cancer
Source: BMC Genomics. 2014 May 20;15(Suppl 4):S2. doi: 10.1186/1471-2164-15-S4-S2 (PMC4083408; doi:10.1186/1471-2164-15-S4-S2)
Supplement: Additional file 2 — The list of interactions in the IL-10 protein-protein interaction network [file 1471-2164-15-S4-S2-S2.docx]

Table S2. The list of interactions in the IL-10 protein-protein interaction network

| PDGFA - PDGFB | UCN3 - IL10RB | A2M - PAEP | UBC - HSPA5 | A2M - TP63 |
| --- | --- | --- | --- | --- |
| IL10 - IL10RA | A2M - APOE | IL10RB - IL10RA | IL10 - IL10RB | HSPA5 - TP63 |
| UBC - TP63 | A2M - APP | A2M - SPACA3 | IL10 - SIRPG | SHBG - A2M |
| UBC - ERBB4 | CD47 - SIRPG | UBC - APOE | APP - LRP1 | IL1B - MMP2 |
| ERBB4 - SIRPG | A2M - LYZ | A2M - LEP | A2M - LRP1 | UBC - CTSB |
| JAK2 - UBC | A2M - MMP2 | IL10 - A2M | A2M - KLK3 | ANXA6 - UBC |
| UBC - APP | HSPA5 - A2M | A2M - CPB2 | APOE - LRP1 | BTRC - UBC |
| A2M - AMBP | CTSE - A2M | A2M - IL1B | IL22 - IL-10RB | A2M - KLK2 |
| APP - APOE | PDGFB - LRP1 | CTSB - AMBP | IGLL5 - SIRPG | IL28A - IL10RB |
| JAK1 - IL10RA | A2M - KLK13 | CTSB - APOE | UBC - LYZ | A2M - MYOC |
| B2M - A2M | IL10RB - UCN2 | A2M - ADAM19 | JAK2 - IL10RA | PDGFA - A2M |
| A2M - ADAMTS1 | TGFBI - A2M | NGF - A2M | IL28B - IL10RB | UBC - ADAM19 |
| A2M - IL4 | F3 - SIRPG | UBC - IL10RA | ANXA6 - A2M | BTRC - TP63 |
| BTRC – IL10RA | CTSB - A2M | A2M - LCAT | PDGFB - A2M | A2M - CELA1 |
